# DEAD-Box Helicase Proteins Disrupt RNA Tertiary Structure Through Helix Capture

**DOI:** 10.1371/journal.pbio.1001981

**Published:** 2014-10-28

**Authors:** Cynthia Pan, Jeffrey P. Potratz, Brian Cannon, Zachary B. Simpson, Jessica L. Ziehr, Pilar Tijerina, Rick Russell

**Affiliations:** Department of Molecular Biosciences and the Institute for Cellular and Molecular Biology, University of Texas at Austin, Austin, Texas, United States of America; Brandeis University, United States of America

## Abstract

DEAD-box helicase proteins accelerate folding and rearrangements of highly structured RNAs and RNA–protein complexes (RNPs) in many essential cellular processes. Although DEAD-box proteins have been shown to use ATP to unwind short RNA helices, it is not known how they disrupt RNA tertiary structure. Here, we use single molecule fluorescence to show that the DEAD-box protein CYT-19 disrupts tertiary structure in a group I intron using a helix capture mechanism. CYT-19 binds to a helix within the structured RNA only after the helix spontaneously loses its tertiary contacts, and then CYT-19 uses ATP to unwind the helix, liberating the product strands. Ded1, a multifunctional yeast DEAD-box protein, gives analogous results with small but reproducible differences that may reflect its *in vivo* roles. The requirement for spontaneous dynamics likely targets DEAD-box proteins toward less stable RNA structures, which are likely to experience greater dynamic fluctuations, and provides a satisfying explanation for previous correlations between RNA stability and CYT-19 unfolding efficiency. Biologically, the ability to sense RNA stability probably biases DEAD-box proteins to act preferentially on less stable misfolded structures and thereby to promote native folding while minimizing spurious interactions with stable, natively folded RNAs. In addition, this straightforward mechanism for RNA remodeling does not require any specific structural environment of the helicase core and is likely to be relevant for DEAD-box proteins that promote RNA rearrangements of RNP complexes including the spliceosome and ribosome.

## Abstract 2

Single-molecule fluorescence experiments reveal how DEAD-box proteins unfold structured RNAs to promote conformational transitions and refolding to the native functional state.

## Author Summary

In addition to carrying genetic information from DNA to protein, RNAs function in many essential cellular processes. This often requires the RNA to form a specific three-dimensional structure, and some functions require cycling between multiple structures. However, RNAs have a strong propensity to become trapped in nonfunctional, misfolded structures. Due to the intrinsic stability of local structure for RNA, these misfolded species can be long-lived and therefore accumulate. ATP-dependent RNA chaperone proteins called DEAD-box proteins are known to promote native RNA folding by disrupting misfolded structures. Although these proteins can unwind short RNA helices, the mechanism by which they act upon higher order tertiary contacts is unknown. Our current work shows that DEAD-box proteins capture transiently exposed RNA helices, preventing any tertiary contacts from reforming and potentially destabilizing the global RNA architecture. Helix unwinding by the DEAD-box protein then allows the product RNA strands to form new contacts. This helix capture mechanism for manipulation of RNA tertiary structure does not require a specific binding motif or structural environment and may be general for DEAD-box helicase proteins that act on structured RNAs.

## Introduction

Structured RNAs are involved in many essential biological processes such as pre-mRNA splicing, regulation of gene expression, protein synthesis, and maintenance of chromosome ends [1]–[5]. These functions require the RNAs to fold into specific structures and, for some, to transition between functional conformations. However, RNAs have a strong propensity for misfolding, and because RNA structure is inherently stable, even at the local level, resolution of misfolded RNAs or rearrangements of structured RNAs can be slow on the biological timescale. These properties suggest a general requirement for RNA folding chaperones *in vivo* [6], and diverse proteins have been shown to possess ATP-dependent or ATP-independent RNA chaperone activity [7],[8].

DEAD-box proteins are superfamily 2 RNA helicases that can function as RNA chaperones to promote the formation and remodeling of functional RNAs and RNPs [9],[10] and are linked to essentially all RNA metabolic processes in all three branches of life [10]–[12]. They use a conserved helicase core of two RecA-like domains to perform a broad range of activities including protein displacement from RNA [13], RNA structure formation [14],[15], and their hallmark activity, ATP-dependent unwinding of short RNA helices [10],[16],[17], including those within structured RNAs [17]. However, in addition to the helical segments that constitute RNA secondary structure, structured RNAs typically include tertiary contacts that must be disrupted during many remodeling processes [18]–[21]. Although it has been proposed that regulated binding to single-stranded RNA (ssRNA) might be sufficient to accelerate disruption of tertiary contacts [22], such disruptions have not been demonstrated for any DEAD-box protein, leaving the mechanisms of these RNA remodeling reactions unclear.

CYT-19, a DEAD-box protein found in the mitochondria of *Neurospora crassa*, functions as a general RNA chaperone [23], facilitating correct folding of diverse group I intron RNAs by accelerating unfolding of misfolded intermediates [17],[19],[24]. Here, we probe how CYT-19 promotes unfolding of structured intermediates by monitoring changes in the secondary and tertiary structure of the P1 helix within the *Tetrahymena thermophila* group I intron ribozyme, which has been extensively studied as an isolated tertiary folding event within a globally folded RNA [25],[26]. The P1 helix forms by base pairing of the ribozyme with an oligonucleotide substrate that mimics the 5′ splice site. This helix docks into tertiary contacts with the ribozyme core, principally through hydrogen bonds between 2′-OH groups within the helix and nucleotides within the core [27]. CYT-19 can unwind the P1 helix, and previous results have shown that the unwinding efficiency depends on the docking stability of the P1 helix, suggesting that unwinding requires loss of the tertiary contacts prior to or during unwinding [17]. However, it was unclear how CYT-19 accomplished the RNA tertiary unfolding and whether it resulted from a known or a novel activity.

To dissect this multistep remodeling process, we used a single molecule Förster resonance energy transfer (smFRET) approach to observe CYT-19 disruption of the 11-bp P1 helix. We directly monitored changes in both tertiary structure and secondary structure, allowing us to independently resolve and quantify the effects of CYT-19 on each step. Thus, we generated a detailed view of the process by which a DEAD-box protein can promote local unfolding of a structured RNA with disruption of tertiary and secondary contacts. Our results lead to a simple physical model that explains previous results, suggests a general mechanism for directing DEAD-box proteins to misfolded RNA intermediates, and is likely to be used broadly by DEAD-box proteins that remodel structured RNAs.

## Results

**Figure 1:**
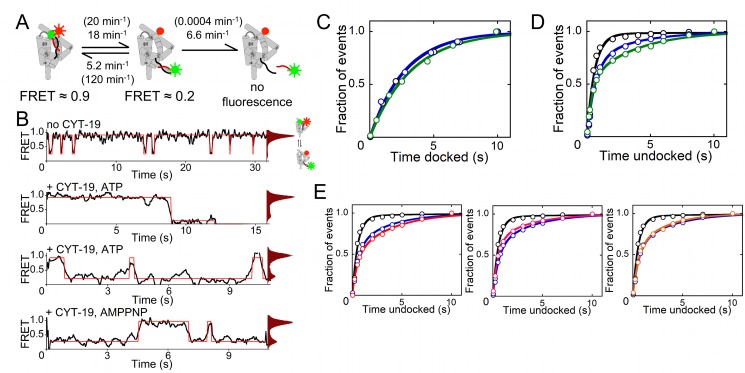
CYT-19 destabilizes tertiary docking of the P1 helix into the *Tetrahymena* ribozyme core. (A) Cartoon of the ribozyme showing P1 helix docking, undocking, and unwinding rate constants in the presence of CYT-19, with the corresponding rate constants without CYT-19 in parentheses (Table S1). (B) Representative FRET traces and histograms showing reversible docking (transitions shown in red) without CYT-19 (top), with CYT-19 and ATP (middle traces), and with CYT-19 and AMP–PNP (bottom). (C and D) Lifetime distributions of the docked (C) and undocked (D) states without CYT-19 (black) or with 0.5 µM (blue) or 1 µM (green) CYT-19 and 2 mM ATP-Mg^2+^ (Figures S2, Tables S1, S2, and Data S1, S2). (E) Lifetime distributions of undocked P1 in the presence of 2 µM CYT-19 with AMP–PNP (red, left plot), without nucleotide (pink, center plot), and with ADP (orange, right plot). In each plot, corresponding data in the absence of CYT-19 and for 2 µM CYT-19 with ATP are shown for comparison in black and blue, respectively (Table S1 and Data S1).

To measure secondary and tertiary transitions of the P1 helix, we used a smFRET system that was designed previously [25],[28],[29]. The ribozyme was extended from its 3′ end and annealed to a complementary oligonucleotide that was immobilized on a microsope slide by a biotin/streptavidin linkage for visualization using total internal reflection (TIR) microscopy (see Materials and Methods and Table S3). Cy3 and Cy5 dyes were positioned such that docked P1 gives efficient energy transfer from Cy3 to Cy5 and a correspondingly high FRET value (∼0.9), whereas undocked P1 gives a greatly reduced FRET value (∼0.2; Figure 1A) [25],[28],[29]. Loss of secondary structure in P1—that is, unwinding of the helix—is detected as a loss of all fluorescence, because the Cy3-labeled strand is released into solution. A loss of fluorescence can also reflect Cy3 photobleaching, which was measured separately and subtracted (Figure S1 and Text S1, “P1 Unwinding Monitored Using Single Molecule Fluorescence and Determination of P1 Docking and Undocking Kinetics”).

### CYT-19 Does Not Accelerate Loss of Tertiary Contacts Between the P1 Helix and the Ribozyme Core

In the absence of CYT-19, P1 was predominantly docked in most molecules but underwent cycles of undocking and redocking, as observed previously under similar conditions [25],[28]. Docking and undocking rate constants were determined from the lifetimes of the P1 helix in the undocked and docked state, respectively, giving a docking rate constant of 120 min^−1^ and an undocking rate constant of 20 min^−1^ (Figure 1B, top trace, Figure 1C–D, Figure S2, and Table S1). Spontaneous unwinding of the P1 helix, as measured by a loss of Cy3 fluorescence beyond the rate expected for photobleaching, was not detectable. However, addition of CYT-19 and ATP led to robust unwinding (Figure 1B, second trace, Figure S3, and Table S1). We found that P1 unwinding occurred primarily from the low FRET state (see Text S1, “P1 Unwinding Monitored Using Single Molecule Fluorescence”). Thus, the CYT-19–mediated remodeling process occurs in two steps, with tertiary undocking preceding helix unwinding. Strikingly, the rate of P1 undocking was not increased (Figure 1C), even with CYT-19 concentrations that approached saturation (see below) and gave substantial increases in the overall unwinding rate (Figure S3 and Table S1). Thus, CYT-19 apparently “waits” for spontaneous loss of the tertiary contacts and then interacts with the undocked P1 helix to unwind it.

### CYT-19 Captures the Undocked P1 Helix, Preventing Redocking

Although CYT-19 does not actively disrupt the P1 docking contact, we found that it increased the lifetime of the P1 helix in the undocked state. In the presence of CYT-19, a substantial fraction of undocked events had long lifetimes, resulting in a slow phase with an observed rate constant for redocking of 20 min^−1^ (Figure 1D). Other undocked events were followed by rapid redocking with the intrinsic docking rate constant (120 min^−1^, Figure 1D), presumably because CYT-19 was not bound or was not positioned to interact with the P1 helix. Supporting a contribution from incomplete binding, the fraction of undocked events with long lifetimes increased with CYT-19 concentration (Table S1), and additional experiments indicated that CYT-19 was approaching saturation at these concentrations but not fully saturated (Figure S3).

For the long-lived undocked complexes, we observed a competition between alternative fates. For undocked events that were not truncated by the termination of data collection, the P1 helix was either unwound, resulting in a loss of fluorescence (56% of events), or it redocked into the ribozyme core (Figure 1B, middle traces, and Figure S4). We calculated unwinding and docking rate constants from the lifetime distributions of these complexes and the probabilities of the alternative outcomes, and we found that CYT-19 slows P1 docking by ∼20-fold to 5.2±2.1 min^−1^ (Tables S1 and S2, and see Text S1, “Determination of P1 Docking and Undocking Kinetics”).

We considered the possibility that CYT-19 might be able to accelerate tertiary unfolding of a helix that forms tertiary contacts less strongly. Thus, we tested two versions of the P1 helix that include specific 2′-methoxy groups shown previously to weaken docking [25],[26],[30]. Although these substitutions increased the rate of undocking in the absence of CYT-19, as expected [25], CYT-19 did not accelerate undocking of the helices (Figure S5 and Tables S1 and S3). Further, CYT-19 retained the ability to capture these helices when they undocked spontaneously, giving decreased rates of redocking that were comparable to that of the standard P1 helix (Figure S5 and Tables S1 and S3).

Together, the results indicate that CYT-19 interferes with P1 docking by binding and capturing the P1 helix after it undocks spontaneously. This “helix capture” mechanism allows CYT-19 to destabilize tertiary docking of the P1 helix, shifting the equilibrium toward the undocked state, without actively disrupting the tertiary contacts.

### ATP Is Not Required for P1 Helix Capture by CYT-19

To probe the role of ATP in CYT-19–mediated destabilization of P1 tertiary docking, we monitored P1 docking behavior with ATP analogs and in the absence of nucleotide. We found that upon replacing ATP with the ATP analog AMP–PNP, ADP, or in the absence of nucleotide, CYT-19 does not unwind the P1 helix significantly, but it retains the ability to block tertiary docking (Figure 1B, bottom trace, Figure 1E, and Table S1). With AMP–PNP, the redocking rate is the same within error as with ATP, whereas the rate is modestly increased with ADP or in the absence of nucleotide (2–3-fold, Table S1). Overall, the lack of a requirement for nucleotide binding suggests that helix capture by CYT-19 does not require closure of the two RecA-like domains of the helicase core [31]–[33] and may result primarily from interactions of the helix with domain 2 (see Discussion) [34].

### Helix Unwinding Can Be Limited by the Rate of Tertiary Contact Disruption

**Figure 2:**
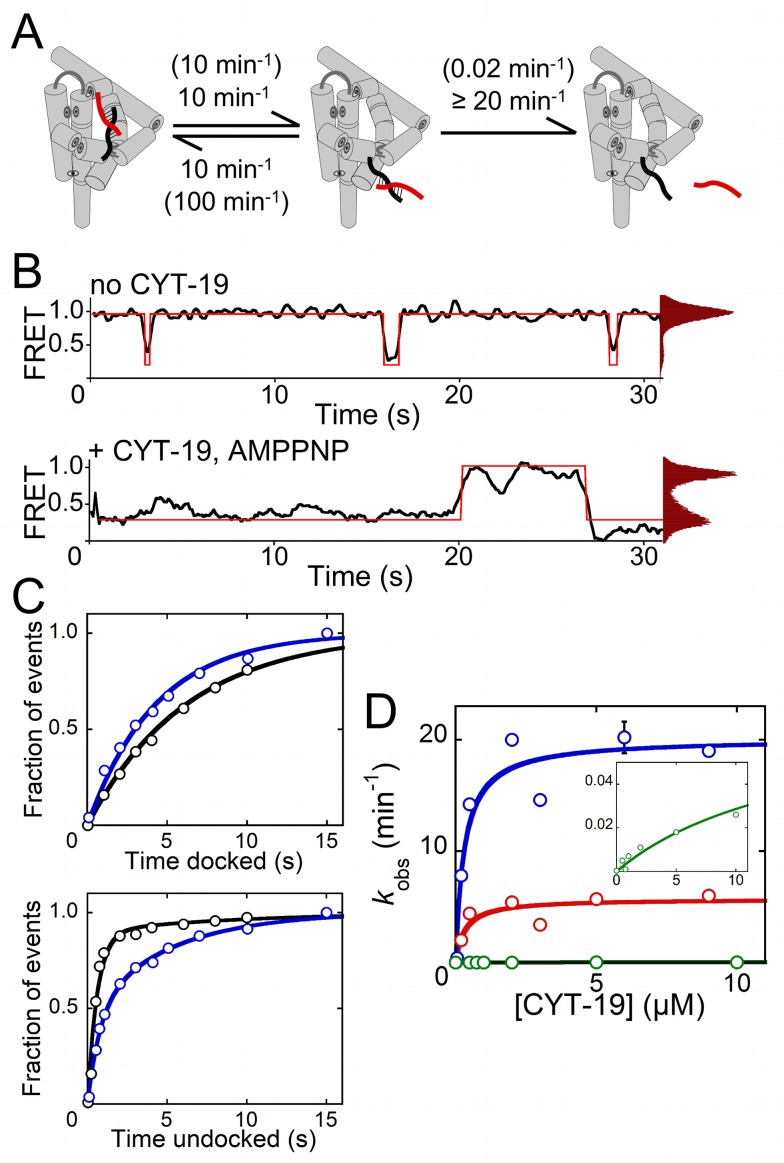
CYT-19–mediated unwinding of a shorter P1 helix (6 bp) is rate limited by spontaneous loss of tertiary contacts. (A) Cartoon representation showing docking, undocking, and unwinding rate constants for the 6-bp P1 helix in the presence of CYT-19. Rate constants in the absence of CYT-19 are shown in parentheses and are similar to previous values [25]. (B) Representative FRET traces and histograms (transitions shown in red) in the absence of CYT-19 (top) and with 1 µM CYT-19 and AMP–PNP (bottom). (C) Lifetime distributions of the docked (top) and undocked (bottom) states in the absence of CYT-19 (black, 102 molecules; Data S1) and with 1 µM CYT-19 and AMP–PNP (blue, 163 molecules; Data S1). (D) CYT-19 unwinding of the P1 helix monitored by ensemble techniques. The maximum observed unwinding rate constant (*k*_max_) for the standard 6-bp P1 helix is 6 min^−1^ (red). Weakening P1 docking by atomic mutagenesis (blue, −3 m, rSA_5_) increases *k*_max_ to 20 min^−1^, and strengthening the docking contacts (green, rP, also in inset) decreases *k*_max_ to 0.075 min^−1^ (Figure S6 and Table S3). Error bars represent the standard deviation of at least two independent measurements.

When CYT-19 interacts with the 11-bp P1 helix, helix unwinding is partially rate limiting for the overall disruption process, as indicated by the substantial fraction of long-lived undocking events that result in P1 redocking rather than unwinding (Table S2). Most helical segments in structured RNAs are shorter than 11 bp and correspondingly less stable, such that unwinding of these helices may be fast enough that the overall process is fully rate limited by the intrinsic loss of the tertiary contacts. We tested this idea using a ribozyme construct with a shorter P1 helix of 6 bp, which also displayed extended undocked lifetimes in the presence of CYT-19 and AMP–PNP (Figure 2A–C). This helix was indeed unwound much faster by CYT-19 in the presence of ATP [17], which precluded generating robust statistics with smFRET (Table S4). Therefore, we used rapid quench-flow techniques to measure the maximum rate constant for the overall process of P1 unwinding by CYT-19 (*k*_max_, which includes loss of tertiary structure and secondary structure). When binding of CYT-19 is saturated, the 6-bp P1 helix was unwound with a *k*_max_ of ∼6 min^−1^, which is comparable to the intrinsic undocking rate constant for this helix, suggesting rate-limiting undocking (Figure 2C–D). As expected from the model, the *k*_max_ value increased when docking was weakened and decreased when docking was strengthened (Figure 2D and Figure S6). Thus, unwinding of a short helix is indeed rate limited by loss of the tertiary interactions, and this tertiary disruption is not accelerated by CYT-19.

### CYT-19 Can Remain Associated with the Ribozyme for Multiple Cycles of Helix Capture

We next used the CYT-19–dependent destabilization of P1 docking to monitor the lifetime of the DEAD-box protein interaction with the ribozyme, testing whether CYT-19 remains associated with the ribozyme after it releases the P1 helix. We were particularly interested in this question because previous work suggested that CYT-19 can form two distinct interactions with RNA simultaneously: one interaction through the helicase core and a second interaction through a highly basic and unstructured “tail” of 50 amino acids (the C-tail) [35],[36]. Thus, it would be possible that an interaction of the C-tail with the ribozyme could persist when the P1 helix is released from the helicase core of CYT-19.

To measure CYT-19 dissociation, we added CYT-19 and AMP–PNP to immobilized ribozyme, and then we washed CYT-19 out of the sample channel so that its dissociation from the ribozyme would be irreversible. We then monitored the FRET values of ribozyme molecules for which the P1 helix was undocked at the start of the observation period following the washout (i.e., those with a low FRET value of ∼0.2). From this collection of molecules, we plotted the average FRET value as a function of time. We interpreted the data in the context of the predictions from two models. In the first model, dissociation of the helicase core from P1 results in dissociation of CYT-19 from the ribozyme. This model predicts that the average FRET value would increase back to the value of 0.85, which reflects the “intrinsic” docking equilibrium of the ribozyme, with a rate constant of ∼5.2 min^−1^, the redocking rate constant for the P1 helix after being captured by CYT-19 (Figure 1A). In the second model, when P1 is released from the helicase core and redocks into the ribozyme core, CYT-19 can remain bound, presumably through its C-tail, so that it can capture P1 when it undocks again. This model would predict a time dependence consisting of at least two exponential phases. An initial increase would reflect the re-equilibration of P1 docking, with CYT-19 remaining bound, and would thus have a rate constant corresponding to the sum of the docking and undocking rate constants with bound CYT-19 (∼23 min^−1^). This phase would be followed by one or more slower phases reflecting CYT-19 dissociation, which would ultimately allow the docking equilibrium to return to its intrinsic state as above.

**Figure 3:**
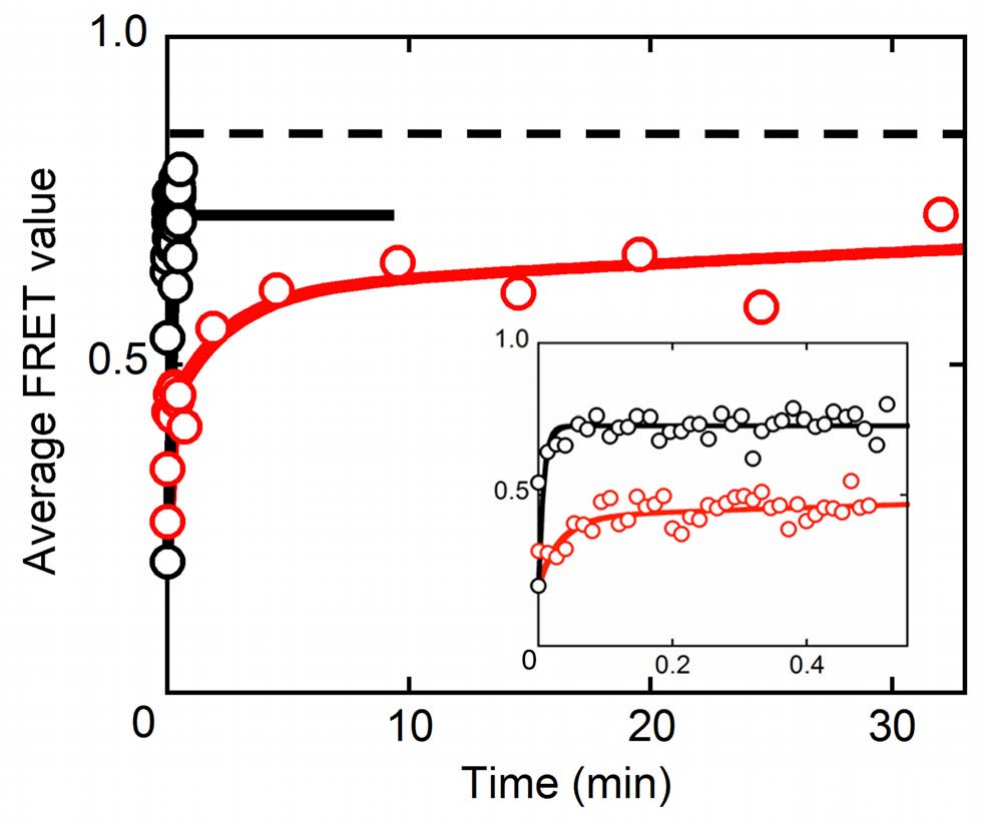
CYT-19 dissociation from the ribozyme. Following a CYT-19 washout in the continued presence of 2 mM AMP–PNP, the average FRET value was followed for ribozyme molecules that started this observation period with the P1 helix undocked (Data S1). The time evolution of the average FRET value for these molecules (red, 62 molecules) was fit by three phases with rate constants and relative amplitudes of 30 min^−1^ (0.36), 0.43 min^−1^ (0.29), and 0.01 min^−1^ (0.35). We infer that the rate constant of 0.43 min^−1^ reflects CYT-19 dissociation because this phase was not observed in the absence of CYT-19. The initial fast phase reflects P1 docking re-equilibration with bound CYT-19 and is predicted from the model, and the slowest phase most likely reflects the slow conversion of ribozyme molecules that initially give poor docking or are misfolded (see Results, “CYT-19 Can Remain Associated with the Ribozyme for Multiple Cycles of Helix Capture”). In the absence of CYT-19 (black, 64 molecules), re-equilibration of P1 docking gave a single observed phase of 130 min^−1^ (inset). The endpoint is lower (0.73) than expected (0.85, indicated by dashed line), most likely reflecting molecules that dock P1 poorly as above.

As predicted by both models, the average FRET value of these molecules increased over time, ultimately returning to a value that reflects the intrinsic P1 docking equilibrium. In strong support of the second model described above, the initial increase in FRET in the presence of CYT-19 occurred with a rate constant of ∼30 min^−1^, which we infer reflects the re-equilibration of P1 docking, whereas CYT-19 remains bound to the ribozyme. A subsequent increase in the average FRET value gave a rate constant of 0.43 min^−1^. This slow phase was not present in a control reaction lacking CYT-19, which gave a single rate constant that reflects rapid P1 redocking (∼130 min^−1^; Figure 3, black). Thus, the slower increase in average FRET value most likely reflects dissociation of CYT-19 from the ribozyme. A very slow third phase was also observed, which most likely reflects slow re-equilibration of ribozyme molecules that form alternative states that dock P1 weakly (see also Figure S2) [28]. In the absence of CYT-19, we did not collect data at the long observation times necessary to measure this phase, but we infer that it was present because the observed endpoint was lower than the expected value (0.73 versus 0.85 expected; Figure 3). Thus, the key conclusion is that CYT-19 can remain bound to the ribozyme after releasing the captured P1 helix. The continued binding, which is most likely mediated through the C-tail of CYT-19, is expected to allow CYT-19 to participate in multiple cycles of helix capture and unwinding, with the helicase core likely remaining poised to capture P1 or other helical elements as they become exposed by transient fluctuations.

### The DEAD-Box Protein Ded1 Also Uses a Helix Capture Mechanism

**Figure 4:**
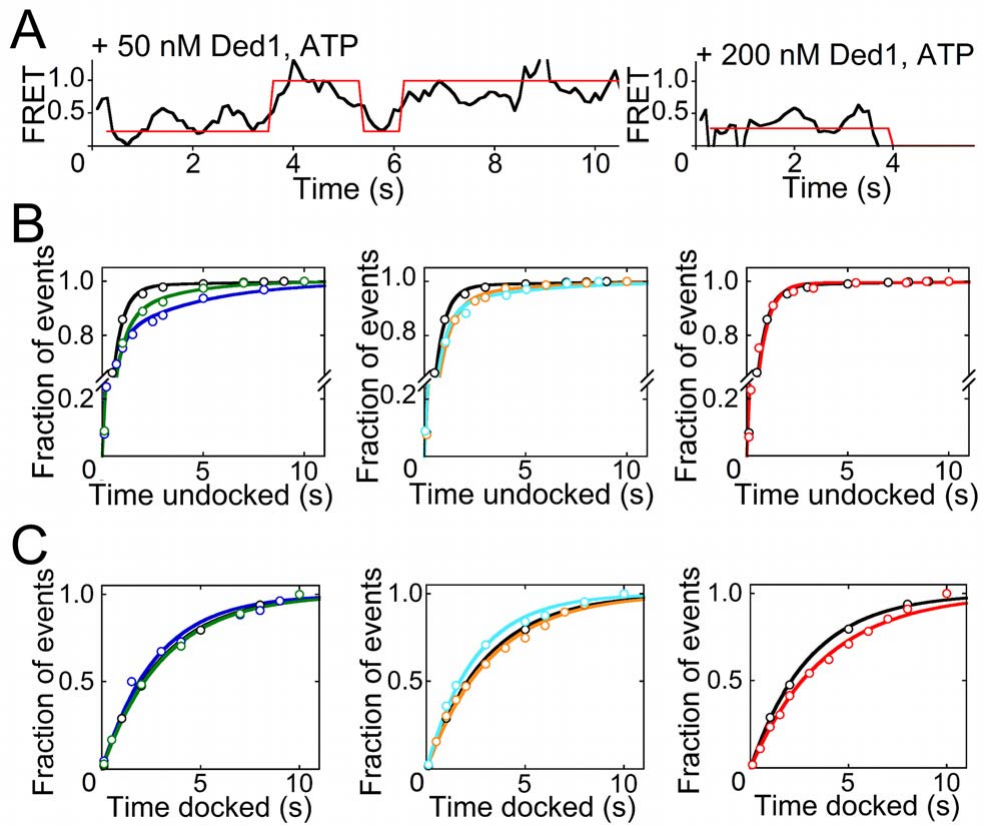
Ded1 destabilizes docking of the P1 helix. (A) Representative FRET traces showing extended undocked lifetimes before redocking (left) and unwinding (right) in the presence of Ded1 and ATP (transitions shown in red). (B) Lifetime plots of the undocked states in the absence of Ded1 (black, all panels), with 50 nM (blue) or 0.2 µM (green) Ded1 and 2 mM ATP (left panel), with 0.1 µM (cyan) or 0.9 µM (orange) Ded1 and 2 mM AMP–PNP (center panel), and with 0.9 µM Ded1 and no nucleotide (red, right panel). (C) Lifetime plots of the docked state of P1 under the same conditions and represented by the same color scheme as (B). See also Data S1. The calculated *k*_dock_ and *k*_undock_ values for each condition are listed in Table S5.

We tested the generality of the helix capture mechanism by using Ded1, a multifunctional DEAD-box protein from *Saccharomyces cerevisiae* [37]–[39]. In the presence of ATP or AMP–PNP, we found that Ded1 uses the same basic mechanism to destabilize tertiary docking of the P1 helix. Specifically, Ded1 does not accelerate the loss of tertiary contacts but slows their subsequent formation (Figure 4 and Table S5), indicating that like CYT-19, Ded1 captures the P1 helix after spontaneous undocking. There are also some interesting differences. First, long-lived undocking of P1 was observed in the presence of ATP or AMP–PNP but not in the absence of nucleotide (Figure 4B, right, and Table S5), indicating that helix capture by Ded1 depends on bound nucleotide. Second, the fraction of P1 undocking events that resulted in helix capture is lower than for CYT-19 and did not depend strongly on Ded1 concentration (Figure 4B, left and center, and Table S5), suggesting that Ded1 is saturating in our experiments for the binding that is responsible for helix capture. However, ensemble unwinding assays display increases in rate constant across the same concentration range (Figure S7). Previous studies have indicated complexity in RNA binding and unwinding by Ded1, with participation of multiple Ded1 protomers [40],[41], which may contribute to the differences we observe between CYT-19 and Ded1 (see Discussion). Despite these differences, Ded1 shares the basic behaviors delineated for CYT-19, capturing the transiently exposed RNA helices and preventing re-formation of tertiary contacts.

## Discussion

**Figure 5:**
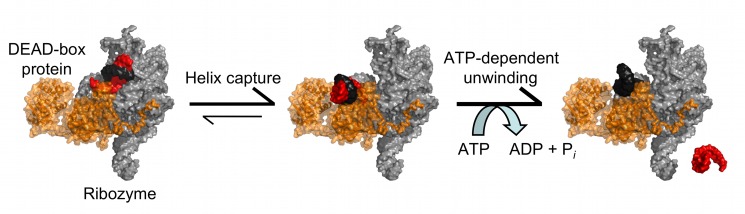
Model for RNA tertiary structure disruption by helix capture. DEAD-box proteins (orange) associate with structured RNAs nonspecifically (left), which can result in the helicase core being positioned to interact with transiently exposed helices (center). This interaction prevents reformation of tertiary contacts by the bound helix, destabilizing the RNA tertiary structure and allowing DEAD-box proteins to use ATP to perform helix unwinding (right). The DEAD-box protein illustrated is the yeast ortholog of CYT-19, Mss116 (pdb 3I5X), and the *Tetrahymena* ribozyme shown is a model structure presented in [62].

Although DEAD-box proteins have previously been shown to promote conformational transitions of highly structured RNAs, which can require extensive disruption of tertiary interactions, it was not known how they disrupt RNA tertiary structure. Here, we used single molecule fluorescence to dissect an RNA unfolding process into discrete steps involving losses of tertiary and secondary structure. Together, our results suggest a straightforward mechanism by which DEAD-box helicase proteins can disrupt RNA tertiary structure (Figure 5). Even if the protein is pre-associated with the RNA, the helicase core does not actively disrupt tertiary contacts. Instead, it captures RNA helices that become exposed transiently by spontaneous fluctuations. For CYT-19, this helix capture process does not require ATP and may result from RNA binding by just one of the two RecA-like core domains, as closure of the two domains typically requires a bound nucleotide [31]–[33]. Supporting this idea, domain 2 of the *S. cerevisiae* DEAD-box protein Mss116 can bind double-stranded RNA (dsRNA) in the absence of an adenosine nucleotide [34]. Ultimately, closure of the domains and consequent unwinding of the RNA helix permits the ssRNA product strands to form new contacts, allowing refolding to a functional structure or exchange between structures.

This helix capture process is reminiscent of a mechanism described for some processive helicases, termed “passive unwinding,” in which the helicase does not actively disrupt base pairs but instead captures the nucleotides from the terminal base pair upon spontaneous fraying, preventing the base pair from reforming. Processive unwinding can be achieved by this mechanism if the helicase protein repetitively captures the frayed end of the helix while it tracks directionally along one of the strands [42],[43]. As each frayed base pair is successively captured, the loss of base stacking is expected to weaken the adjacent base pair, accelerating its fraying and therefore accelerating unwinding [43]. In a conceptually analogous manner, when a DEAD-box protein captures a helix from a structured RNA, it will not only destabilize tertiary structure by preventing reformation of tertiary contacts by the captured helix, but it will also weaken additional tertiary contacts within the folded RNA if they form cooperatively [44]–[46]. Thus, despite its passive nature, this helix capture mechanism is expected to accelerate the kinetics of large-scale tertiary unfolding of structured RNAs.

This mechanism for unfolding RNA tertiary structure is likely to be used broadly by DEAD-box proteins that function to promote RNA folding, as it relies on their inherent abilities to bind dsRNA and induce ATP-dependent helix unwinding [34], and does not depend on any specific protein binding site or structural context. Previous work showed that CYT-19 can unfold the *Tetrahymena* ribozyme with an efficiency that depends on the overall stability of the RNA [24], and helix capture provides a physical model for this result. Less stable structures are expected to undergo more frequent dynamic fluctuations, allowing for more frequent capture events and therefore more efficient unfolding. Thus, this mechanism allows DEAD-box proteins to sense RNA stability, leading to preferential action on less stable misfolded intermediates, regardless of specific structural features in the misfolded RNAs, while minimizing activity upon stable, natively folded RNA. Consistent with this view, CYT-19 is activated for ATPase activity to a lower extent by the natively folded wild-type *Tetrahymena* ribozyme than by less stable mutants, suggesting fewer productive interactions with the more stable structure [47]. A corollary of the model is that groups of cellular RNAs that lack stable tertiary structure, such as mRNAs, are potentially subject to unfolding by DEAD-box proteins. Indeed, recent work has shown that cellular mRNAs are continually remodeled, such that they are less structured on average than they are under standard *in vitro* conditions [48]. Furthermore, this remodeling requires ATP [48], highlighting the roles of RNA helicase proteins as general manipulators of RNA structure *in vivo*.

To test whether the helix capture mechanism is used by DEAD-box proteins beyond CYT-19, we monitored P1 helix unwinding by the multifunctional yeast protein Ded1. Ded1 is implicated in many processes that involve remodeling of mRNAs and mRNPs, including mRNA splicing [49], transcription initiation [50]–[54] and repression [53],[54], ribosome scanning [55], RNA interference [56],[57], and RNA storage and decay [53],[54]. Our findings that Ded1 does not accelerate P1 undocking and that it slows P1 redocking show that, like CYT-19, Ded1 captures the P1 helix after it loses tertiary contacts spontaneously, thus relying on the same general mechanism for RNA tertiary structure disruption. There are also two notable differences between the proteins. Most strikingly, helix capture by Ded1 requires nucleotide binding, whereas helix capture by CYT-19 does not. One possibility is that helix capture by Ded1 involves closure of the two core domains, in which case the capture event may occur concomitantly with local strand separation [34]. However, any strand separation must be insufficient to give complete unwinding of the P1 helix, because we observe the completion of unwinding as a second, slower step that results in dissociation of the Cy3-labeled oligonucleotide. Alternatively, the nucleotide requirement may reflect a difference in the RNA binding and unwinding modes of Ded1. Unlike CYT-19, which is thought to use its C-tail as a tether for interaction with structured RNA, Ded1 is thought to function as a multimer, with one or more Ded1 monomers interacting with RNA structures or ssRNA extensions to localize an additional Ded1 monomer that performs helix unwinding [11],[22],[41]. Importantly, the Ded1 that binds the extension and serves as the landing site most likely associates through its helicase core in a nucleotide-dependent manner [11],[22],[41]. Thus, the nucleotide requirement for helix capture may arise not from the Ded1 molecule that interacts directly with P1 but instead from a molecule that binds elsewhere on the ribozyme and recruits the Ded1 protein that binds P1.

A second difference is that Ded1 has a lower helix capture efficiency than CYT-19, even at protein concentrations that appear to be saturating. It is possible that when the helicase core of Ded1 binds a dsRNA, it forms an initial encounter complex that frequently dissociates and is not detected by our method. It is notable that the *in vivo* substrates of Ded1 tend to be less structured than the group I intron substrates encountered by CYT-19 and therefore may not require a robust helix capture efficiency. An alternative explanation is that Ded1 is preferentially positioned on the ribozyme in our single molecule experiments, most likely by additional interactions with a second Ded1 monomer as described above, and this positioning is suboptimal for capturing P1 when it undocks transiently (but close enough to block other Ded1 monomers from solution). In this case, the low capture efficiency may not be a general property of Ded1. Indeed, Ded1 is comparable to CYT-19 in its ability to promote folding transitions of group I introns [58] and at least as active as CYT-19 for overall unwinding of isolated RNA helices [17],[58] and of the P1 helix within the context of the ribozyme ([17] and Figure S7). Although further studies focused on Ded1 will be required to determine the origins of the specific behaviors of Ded1, the work here demonstrates that Ded1 can disrupt RNA tertiary structure using a helix capture mechanism.

In addition to DEAD-box proteins that function as general RNA chaperones, the helix capture mechanism may also be important for DEAD-box proteins that function more specifically in processes such as assembly of the ribosome and spliceosome [59]–[61]. In these processes, capture and unwinding of dynamic helices would be expected to promote conformational transitions, whereas formation of a stable, folded surface would indicate that an RNA folding or protein assembly step has proceeded correctly. Thus, this helix capture mechanism is likely to be used widely by DEAD-box proteins, ranging from those that function as general RNA chaperones to those that promote specific RNA structural transitions in complex biological processes.

## Materials and Methods

### Protein Purification

CYT-19 was purified as previously described (see Text S2, “CYT-19 Purification,” for details) [24].

### Ribozyme Preparation

For ensemble experiments, the L-21/ScaI form of the *T. thermophila* group I ribozyme was prepared by *in vitro* transcription (>4 h at 37°C with 25 mM MgCl_2_) [17]. For single molecule experiments, L-21/T2, a form of the ribozyme that is extended at the 3′-end with the tail sequence ACCAAAAUCAACCUAAAACUUACACA, was prepared under the same conditions [29]. L-16/ScaI, a version of the ribozyme with a 5′-extension of GGUUU (resulting in an 11-bp P1 helix), and L-16/T2, which includes both the 5′- and 3′-extensions, were transcribed *in vitro* at 30°C for 30 min with 4 mM MgCl_2_ to minimize self-cleavage [28]. All RNAs were then purified with RNeasy columns (Qiagen) and stored in TE buffer at −20°C.

Dye-labeled oligonucleotides were purchased from IDT and unlabeled RNA oligonucleotides were purchased from Dharmacon. All oligonucleotides were stored in TE buffer at −20°C. For ensemble experiments, substrate oligonucleotides were 5′-end labeled with [γ^32^-P]ATP (PerkinElmer) using T4 polynucleotide kinase (New England Biolabs). See Table S3 for sequences of all oligonucleotides used.

### Ensemble Unwinding Experiments

Benchtop and rapid quench-flow experiments monitoring the unwinding activity of CYT-19 or Ded1 were performed at 25°C in 50 mM Na-MOPS (pH 7.0), 10 mM MgCl_2_, 50 mM KCl, 2 mM ATP-Mg^2+^ (ATP mixed with an equal amount of MgCl_2_), and 5% glycerol as previously described [17]. Ribozymes were prefolded to the native state in 50 mM Na-MOPS (pH 7.0) and 10 mM MgCl_2_ for 30 min at 50°C [17],[28],[29]. Alternatively, the misfolded ribozyme was generated by incubation in 50 mM Na-MOPS (pH 7.0) and 10 mM MgCl_2_ for 5 min at 25°C [17],[18]. Trace radiolabeled substrate was incubated with prefolded native or misfolded ribozyme for 5 min at 25°C. Unwinding reactions were initiated by adding CYT-19 or Ded1 and at least 25-fold excess unlabeled substrate and quenched to a solution of 33 mM MgCl_2_ and 1 mg/ml Proteinase K. Bound and unbound substrates were separated on a 20% native polyacrylamide gel at 4°C and quantified using a PhosphorImager and ImageQuant (GE Healthcare). Data were analyzed using Kaleidagraph (Synergy Software).

### TIR Fluorescence (TIRF) Microscope

A diode-pumped solid-state green laser (532 nm; CrystaLaser GCL-100-M) and a red laser (637 nm; Coherent, maximum power 50 mW) were directed through a prism at an angle that allows TIR at the surface of the sample channel, which was constructed from a glass cover slip adhered to a quartz slide with double-sided tape. The surfaces of both the cover slip and slide were passivated with a mixture of mPEG and biotin-PEG, allowing for ribozyme immobilization while preventing protein adsorption to the slide surface (see Text S2 for description of slide preparation). Images were collected using a 60× water-immersion Olympus UPlanApo objective (numerical aperture, 1.2), filtered through a 550-nm long-pass filter (Chroma Technology) to remove scattered excitation light, separated into “green” and “red” images using dichroic mirrors, and focused onto the two halves of a microchannel plate intensified charge-multiplying charge-coupled device (CCD) (I-Penta*MAX*, Princeton Instruments, Roper Scientific, Inc.).

### Single Molecule Fluorescence Data Acquisition

The ribozyme was annealed to biotinylated, Cy5-labeled tether (≥10∶1 molar ratio of ribozyme to tether) in 50 mM Na-MOPS (pH 7.0) with 200 mM NaCl by heating at 95°C for 1 min before cooling at 0.1°C/s to 50°C. The ribozyme was then folded to its native conformation by adding MgCl_2_ to a final concentration of 10 mM and incubating the solution at 50°C for 30 min. Cy3-labeled substrate oligonucleotides were then added to the prefolded ribozyme at approximately 7-fold excess and incubated for 5 min at 25°C in ribozyme buffer (50 mM MOPS, pH 7.0, 10 mM MgCl_2_). The ribozyme-substrate-tether complex was then diluted to 10–25 pM in ribozyme buffer and immobilized onto PEG slides via a biotin-streptavidin linkage (see Text S2 for description of slide preparation).

To measure P1 docking and unwinding, various concentrations of CYT-19 or Ded1 protein were diluted in CYT-19 buffer solution (50 mM Na-MOPS, pH 7.0, 10 mM MgCl_2_, 50 mM KCl, 5% glycerol). For some experiments, ATP or another nucleotide (see Table S1) was added to a final concentration of 2 mM. The solution was then flowed through the sample channel along with an oxygen scavenging system (OSS) of 1 mM Trolox [(±)-6-hydroxy-2,5,7,8-tetramethylchromane-2-carboxylic acid, Aldrich,>97%], 500 mM glucose, 0.1 mg/ml glucose oxidase, and 0.06 mg/ml catalase. Images of the dye-labeled molecules within the sample channel were collected in 40-ms or 100-ms frames for 10–30 s (fully intensified at ∼1,000 V).

To measure CYT-19 dissociation, slide-immobilized ribozyme was incubated with near-saturating concentrations of CYT-19 (1–2 µM) along with 2 mM AMP–PNP for at least 2 min at 25°C. The sample channel was then washed with a solution of CYT-19 buffer, AMP–PNP, and OSS to remove the protein from solution, preventing CYT-19 from rebinding. After a dead time of ∼30 s, data recordings were acquired at 2-s frames for 5–10 s (to reduce dye photobleaching) every 30 s over a period of 30 min. Molecules that were present in the low FRET state at the start of data collection were selected to bias the analysis towards protein-bound ribozymes. This is because the fraction of ribozyme molecules that are undocked at given time is low in the absence of CYT-19, whereas a fraction of the protein-bound molecules would be expected to persist in the undocked state during the dead time of 30 s. Fluorescence signals were collected under green laser excitation and then under red laser excitation for colocalization of Cy3 with Cy5. The average signal-to-noise ratio was ∼5, with green laser intensity averaging ∼15 mW (measured near the laser aperture).

### Single Molecule Data Analysis

All relevant data are within the article and its Supporting Information files, except primary data, including raw intensity values for donor and acceptor fluorophores, which are available from the UT Box database (https://utexas.box.com/s/t0va9jj9x2xbf3wilxxg).

## Supporting Information

Figure S1 Measurement of the rate constant for Cy3 photobleaching. A Cy3-labeled oligonucleotide corresponding to the “tether” oligonucleotide (see Table S3) was immobilized on a PEG-treated slide and excited constantly by the green laser (532 nm) at 15 mW. Photobleaching of Cy3 under our experimental conditions (see Materials and Methods) was measured by monitoring the number of molecules that retained Cy3 fluorescence as a function of time (blue, 0.34 min^−1^). Analogous data were collected with 2 µM CYT-19 and AMP–PNP in solution to determine whether these solutes affect photobleaching (red, 0.55 min^−1^).
(TIF)

Figure S2 Representative FRET traces showing heterogeneous P1 docking behavior in the absence of CYT-19. Although most molecules gave behavior as shown in the top FRET trace (>90% of all molecules observed), longer undocked dwell times were observed for some molecules (transitions shown in red). Some of these molecules may be misfolded and therefore not support stable docking of P1 [29]. In addition, conformational heterogeneity in docking behavior has been previously observed for this ribozyme construct in single molecule experiments [28]. As a result of this small population of ribozymes for which the P1 helix does not dock stably (<10%), a minor phase with an increased τ_undocked_ is observed in the absence of CYT-19 (Table S1).
(TIF)

Figure S3 Unwinding of the standard 11-bp P1 helix by CYT-19. Observed rate constants for P1 unwinding determined in ensemble measurements are plotted against CYT-19 concentration (see Materials and Methods). The hyperbolic fit gives a second order rate constant of 1.5×10^5^ M^−1^·min^−1^ with a maximum unwinding rate constant (*k*_max_) of 0.86 min^−1^ and a *K*_1/2_ value of 5.7 µM CYT-19. Analogous single molecule measurements, in which the number of remaining substrate molecules was determined over time from multiple fields of view, gave comparable observed rate constants (within 3–5-fold, Table S1).
(TIF)

Figure S4 From the CYT-19–bound undocked state, the P1 helix can redock into tertiary contacts with the ribozyme core or be unwound by CYT-19. To determine whether these alternative fates arise from a kinetic competition from the same population of undocked molecules or whether they are different populations that are predetermined to undergo one fate or the other, we separately analyzed the lifetimes of P1 undocking events that led to redocking or to unwinding. The corresponding rate constants for events that led to redocking (black, 22 min^−1^) and unwinding (blue, 20 min^−1^) are comparable to each other and to *k*_obs_ when all of the undocked complexes are considered together (20 min^−1^, Figure 1D). Therefore, these results indicate that P1 unwinding and redocking are competing processes that originate from the same initial population of undocked P1.
(TIF)

Figure S5 Effect of CYT-19 on docking of the 11-bp P1 helix of the *Tetrahymena* ribozyme with *K*_dock_∼0.6. (A) Representative FRET traces (transitions shown in red) and corresponding histograms of the docking equilibrium in the absence of CYT-19 (top) and with 1 µM CYT-19 and 2 mM ATP-Mg^2+^ (bottom) for a P1 helix formed with the oligonucleotide −1 m,rSA_3_C_2_ (see Table S3). (B) Lifetime plots for docked and undocked P1 in the absence of CYT-19 (black) and with 1 µM CYT-19 and 2 mM ATP-Mg^2+^ (blue). See also Data S1. Values of the docking rate and equilibrium constants are shown in Table S1 for this helix and a second helix that docks weakly (formed with −3 m,rSA_3_C_2_; see Table S3).
(TIF)

Figure S6 CYT-19–mediated unwinding of the 6-bp P1 helix is rate limited by spontaneous undocking of P1. To verify that the observed correlation between the maximum P1 unwinding rate and the undocking rate is due to P1 docking stability, ensemble experiments were performed with the native *Tetrahymena* ribozyme and its long-lived misfolded conformer, which does not stably dock the P1 helix [29]. See Table S3 for sequences and properties of substrate oligonucleotides. (A) The CYT-19 concentration dependence for unwinding the 6-bp P1 helix formed with substrate −1 d,rSA_5_ by the native ribozyme shows a maximum unwinding rate constant (*k*_max_) of 6 min^−1^ (red), which is comparable to the intrinsic undocking rate constant measured in single molecule experiments (Figure 2C, top and Table S1). When docking is inhibited by misfolding the ribozyme (blue), *k*_max_ is increased to ∼30 min^−1^. (B) With a substrate for which P1 docking is inhibited by replacement of a 2′-hydroxyl group with a 2′-O-methyl group (−3 m,rSA_5_), the undocked state predominates and CYT-19–mediated unwinding is accelerated, with no difference between the native ribozyme (red) and the misfolded ribozyme (blue). We infer that the lower value for the *k*_max_ of this substrate compared to the standard substrate (−1 d,rSA_5_, Figure S6A) reflects an effect of the methoxy substitution on CYT-19–mediated unwinding. (C) CYT-19–mediated unwinding of the P1 duplex containing the 6-nt product (rP), which docks much more strongly than the helix formed with the standard substrate. As above, results from the native and misfolded ribozyme species are shown in red and blue, respectively. Error bars represent the standard deviation of at least two independent measurements.
(TIF)

Figure S7 Unwinding of the standard 11-bp P1 helix by Ded1. Ded1 unwinds P1 in the presence of 2 mM ATP with a second order rate constant of 3.4×10^6^ M^−1^·min^−1^ (black). Secondary structure disruption by Ded1 is reduced in the presence of 2 mM AMP–PNP (orange, 4.6×10^5^ M^−1^·min^−1^), and without nucleotide (red, 2.8×10^3^ M^−1^·min^−1^).
(TIF)

Table S1 P1 docking kinetics and equilibria for the 11-bp P1 helix. Values were determined in single molecule fluorescence experiments except where indicated. The slow phase for P1 docking in the absence of CYT-19 is attributed to heterogeneous P1 docking behavior (Figure S2). The docking rate constant in the presence of CYT-19 (*k*_dock_) was calculated as described in Text S1 (“Determination of P1 Docking and Undocking Kinetics”). Except where indicated, the observed rate constant for unwinding (*k*_obs,unwind_) was determined by single molecule fluorescence by monitoring the disappearance of substrate from the ribozyme over time, using multiple fields of view. Thus, *k*_obs,unwind_ reflects the overall rate constant for the two-step process of undocking and helix unwinding. See Table S3 for sequences and effects of each substrate. See also Data S1. ^a^Relative amplitudes for each phase of the docking kinetics were determined from the fit of the undocked lifetimes normalized by total number of transition events and are listed in parentheses. ^b^Rate constants for P1 unwinding in the absence of CYT-19 were measured in ensemble experiments.
(DOCX)

Table S2 Rate constants of the various “fates” of undocked P1 helix. In the presence of CYT-19, the undocked P1 helix may redock or unwind. Additionally, the fluorescence signal may be artificially truncated by the shuttering of the excitation laser. For each CYT-19 concentration, the fractions of undocking events that ended with redocking, unwinding, or were truncated by the shutter were determined and the corresponding rate constants (*k*_dock_, *k*_unwind_, and *k*_truncation_, respectively) were calculated by multiplying the observed rate constant (*k*_obs_) by the probabilities of each outcome (see Text S1, “Determination of P1 Docking and Undocking Kinetics” for details). To determine the unwinding rate constant (*k*_unwind_), the calculated rate constant reflecting disappearance of Cy3 was further corrected by subtracting the rate constant for Cy3 photobleaching, as measured independently (*k*_photobleach_ = 0.55 min^−1^; Figure S1). Values reported in the text as the fraction of events that ended in unwinding or redocking express these outcomes relative to each other—that is, normalized to 100%.
(DOCX)

Table S3 Sequences and properties of oligonucleotides used in ensemble and single molecule experiments. In order for the P1 helix to be visualized with smFRET, the indicated oligonucleotides were labeled on their 3′-end with Cy3 dye and the DNA tether was labeled with its FRET pair, Cy5. For the first two oligonucleotides, Cy3 replaces the 3′ nucleotide (i.e., resulting in −1 d, rSA_4_-Cy3 and −3 m,rSA_4_-Cy3).
(DOCX)

Table S4 Single molecule observation of *Tetrahymena* ribozyme with the 6-bp P1 before and after addition of 10 nM CYT-19. Prior to CYT-19 addition, each field of view on the slide showed an average of 17 molecules (from three FOVs), as indicated. Upon CYT-19 addition, the number of visible molecules decreased and remained constant, as expected based on the P1 unwinding rate constant measured under the same conditions in ensemble experiments [17]. The number of molecules for each time point shown was determined for different FOVs on the slide to minimize the contribution of dye photobleaching. The low number of ribozyme molecules observed per FOV after the addition of CYT-19 and the lack of a detectable time dependence prevented a robust analysis of the time dependence or docking dynamics for this shorter P1 helix.
(DOCX)

Table S5 Docking kinetics for the 11-bp P1 helix as measured by single molecule fluorescence in the presence of Ded1 and the indicated nucleotides. The docking rate constant in the presence of Ded1 (*k*_dock_) was calculated as for CYT-19 (see Text S1, “Determination of P1 Docking and Undocking Kinetics”). The rate constant for P1 unwinding by Ded1 (*k*_uw_) was also calculated as described for CYT-19 and determined to be 5.5±2.1 min^−1^. See also Data 1. ^a^Amplitudes for each phase of the docking kinetics, listed in parentheses, were determined from the fit of the undocked lifetimes and normalized by the total number of transition events.
(DOCX)

Data S1 Single molecule data underlying the lifetime distribution plots.
(XLS)

Data S2 Representative movie showing the *Tetrahymena* ribozyme with an 11-bp P1 helix in the presence of 1 µM CYT-19 and 2 mM ATP-Mg^2+^. The ribozyme was dye-labeled as described in Text S1 and excited under green laser (for the first ∼12 s of the movie) and then red laser (starting at ∼17 s).
(AVI)

Text S1 Single molecule data analysis, including descriptions of molecule selection, determination of P1 docking and undocking kinetics, and P1 unwinding as monitored by single molecule fluorescence.
(DOC)

Text S2 Supplementary methods, including purification of CYT-19 and slide preparation for single molecule experiments.
(DOC)

## Abbreviations

AMP-PNP: adenosine 5′-(β,γ-imido)triphosphate
bp: base pair
dsRNA: double-stranded RNA
FRET: Förster resonance energy transfer
OSS: oxygen scavenging system
RNP: ribonucleoprotein complex
smFRET: single molecule FRET
ssRNA: single-stranded RNA
