# Supplementary figures and images for: DEAD-Box Helicase Proteins Disrupt RNA Tertiary Structure Through Helix Capture
Source: PLoS Biol. 2014 Oct 28;12(10):e1001981. doi: 10.1371/journal.pbio.1001981 (PMC4211656; doi:10.1371/journal.pbio.1001981)

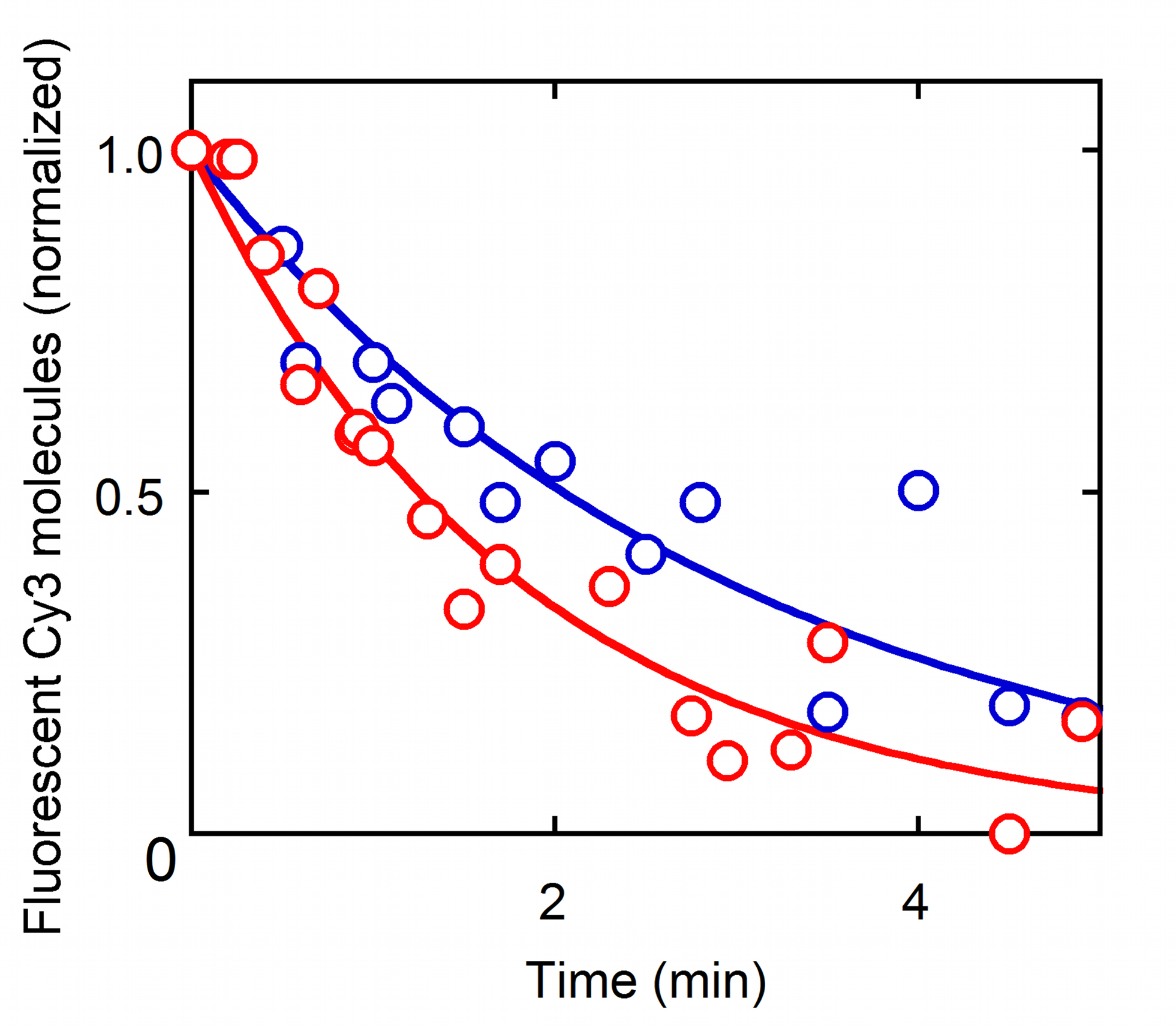

Supplement: Figure S1 — Measurement of the rate constant for Cy3 photobleaching. A Cy3-labeled oligonucleotide corresponding to the “tether” oligonucleotide (see Table S3) was immobilized on a PEG-treated slide and excited constantly by the green laser (532 nm) at 15 mW. Photobleaching of Cy3 under our experimental conditions (see Materials and Methods) was measured by monitoring the number of molecules that retained Cy3 fluorescence as a function of time (blue, 0.34 min−1). Analogous data were collected with 2 µM CYT-19 and AMP–PNP in solution to determine whether these solutes affect photobleaching (red, 0.55 min−1). (TIF) [file pbio.1001981.s001.tif]

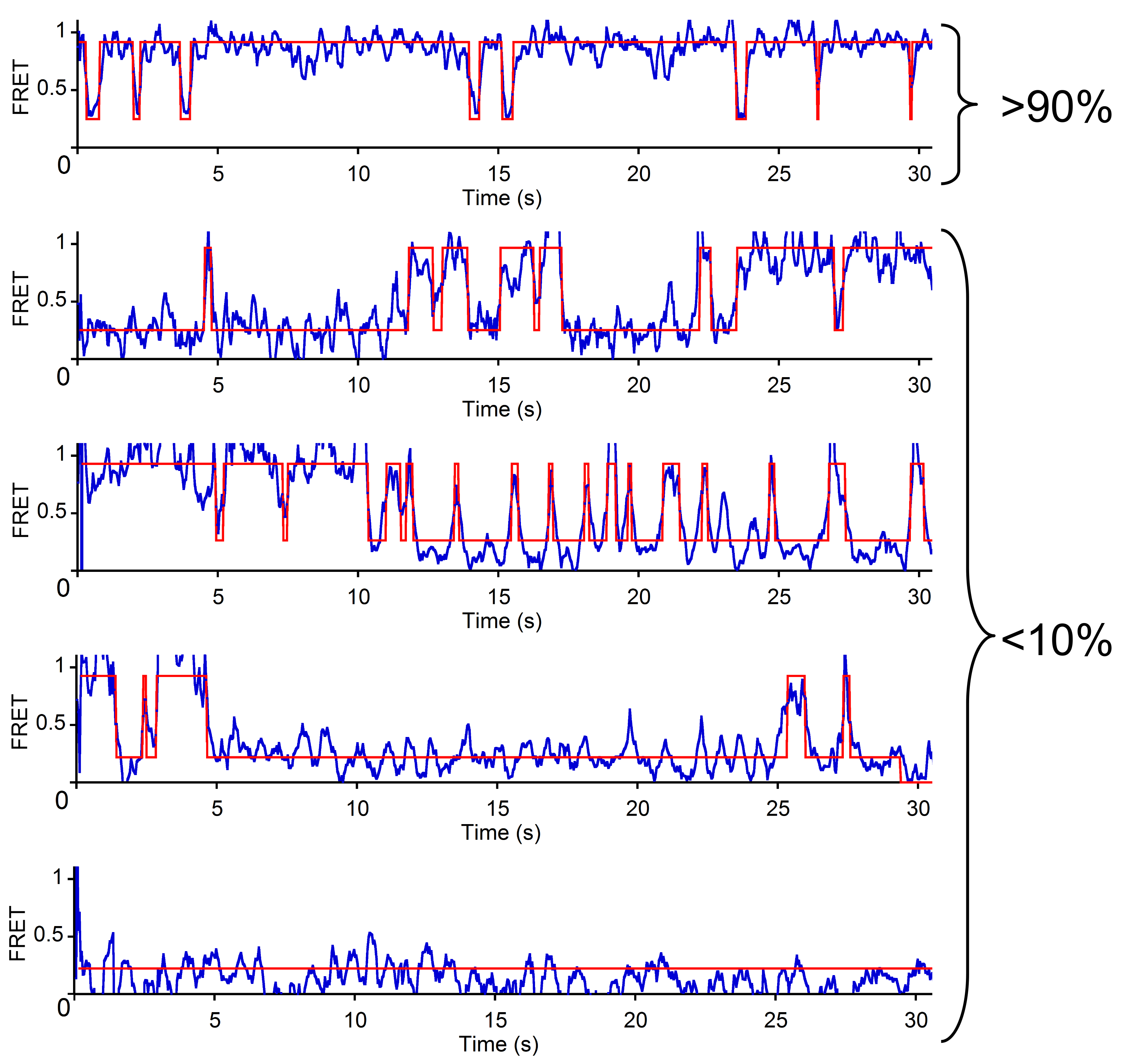

Supplement: Figure S2 — Representative FRET traces showing heterogeneous P1 docking behavior in the absence of CYT-19. Although most molecules gave behavior as shown in the top FRET trace (>90% of all molecules observed), longer undocked dwell times were observed for some molecules (transitions shown in red). Some of these molecules may be misfolded and therefore not support stable docking of P1 [29]. In addition, conformational heterogeneity in docking behavior has been previously observed for this ribozyme construct in single molecule experiments [28]. As a result of this small population of ribozymes for which the P1 helix does not dock stably (<10%), a minor phase with an increased τundocked is observed in the absence of CYT-19 (Table S1). (TIF) [file pbio.1001981.s002.tif]

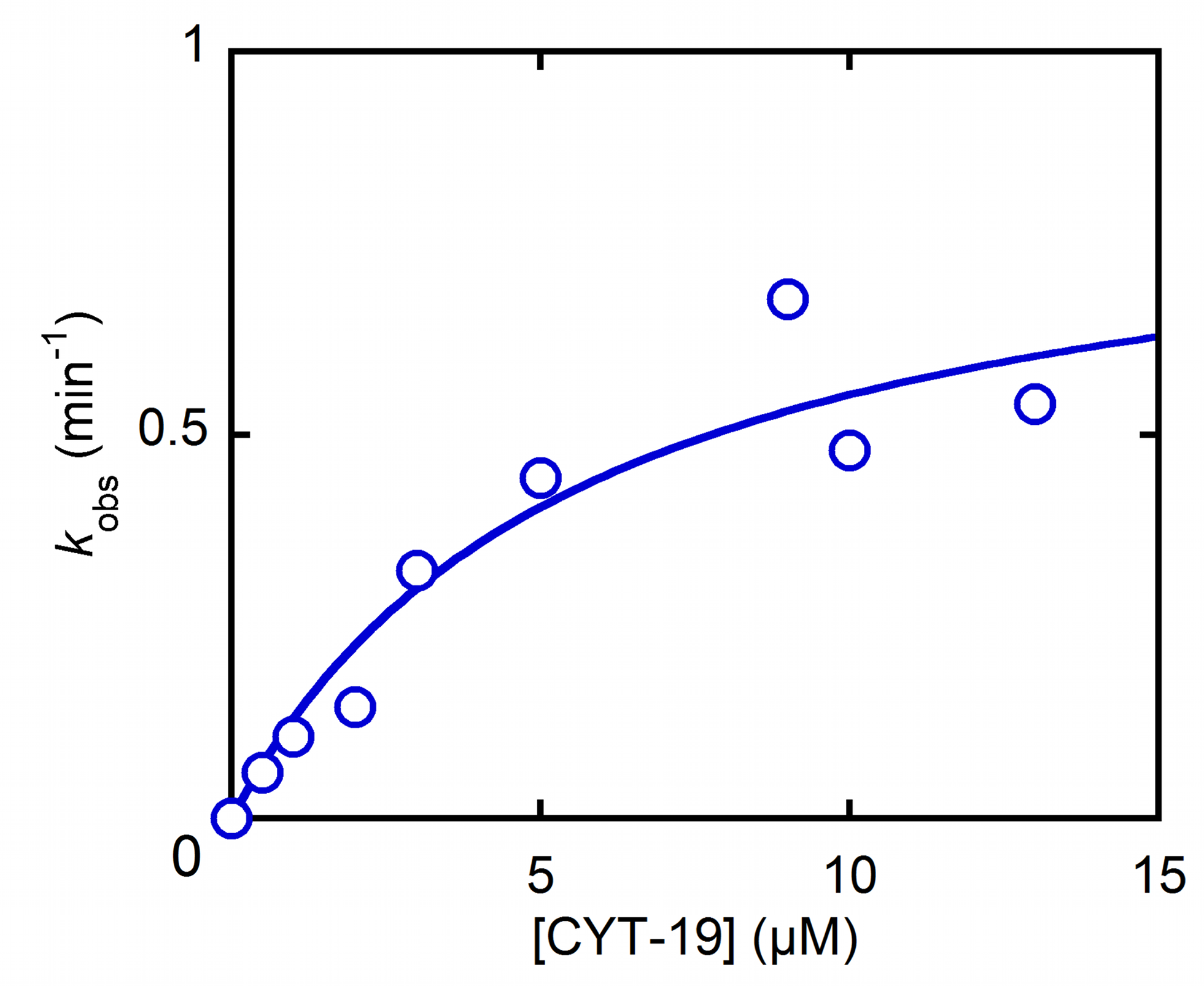

Supplement: Figure S3 — Unwinding of the standard 11-bp P1 helix by CYT-19. Observed rate constants for P1 unwinding determined in ensemble measurements are plotted against CYT-19 concentration (see Materials and Methods). The hyperbolic fit gives a second order rate constant of 1.5×10^5^ M−1·min−1 with a maximum unwinding rate constant (k max) of 0.86 min−1 and a K 1/2 value of 5.7 µM CYT-19. Analogous single molecule measurements, in which the number of remaining substrate molecules was determined over time from multiple fields of view, gave comparable observed rate constants (within 3–5-fold, Table S1). (TIF) [file pbio.1001981.s003.tif]

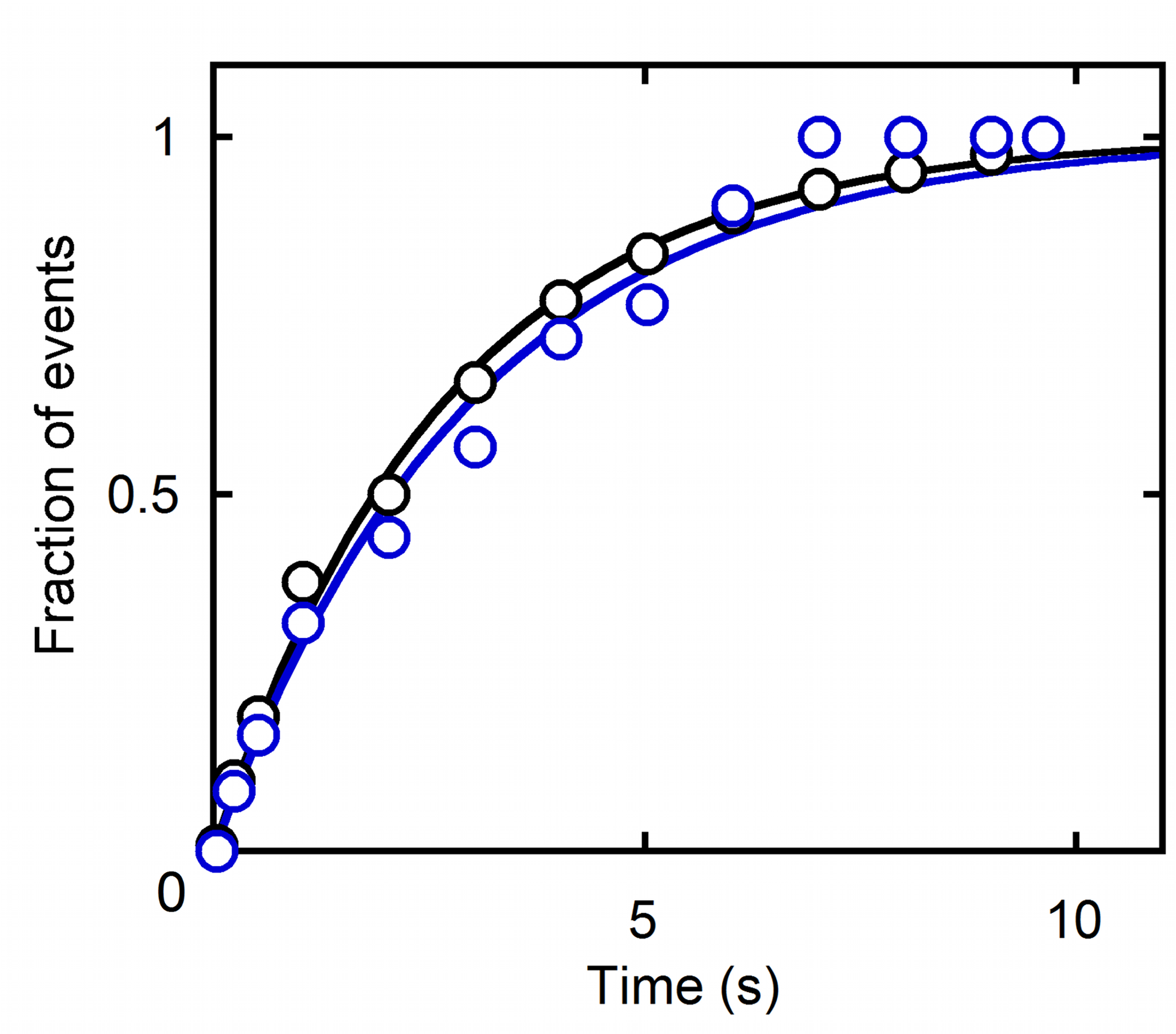

Supplement: Figure S4 — From the CYT-19–bound undocked state, the P1 helix can redock into tertiary contacts with the ribozyme core or be unwound by CYT-19. To determine whether these alternative fates arise from a kinetic competition from the same population of undocked molecules or whether they are different populations that are predetermined to undergo one fate or the other, we separately analyzed the lifetimes of P1 undocking events that led to redocking or to unwinding. The corresponding rate constants for events that led to redocking (black, 22 min−1) and unwinding (blue, 20 min−1) are comparable to each other and to k obs when all of the undocked complexes are considered together (20 min−1, Figure 1D). Therefore, these results indicate that P1 unwinding and redocking are competing processes that originate from the same initial population of undocked P1. (TIF) [file pbio.1001981.s004.tif]

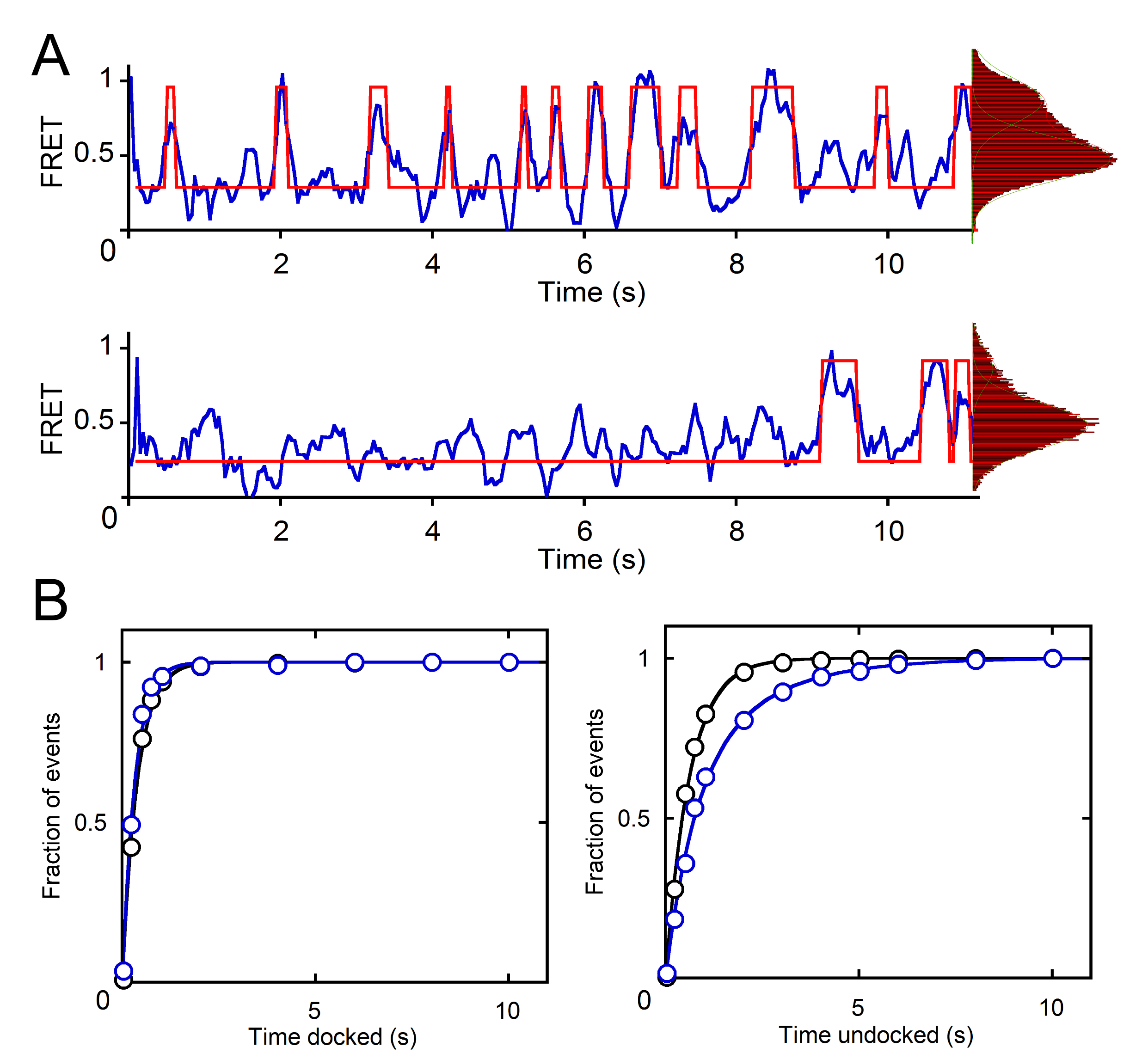

Supplement: Figure S5 — Effect of CYT-19 on docking of the 11-bp P1 helix of the Tetrahymena ribozyme with K dock∼0.6. (A) Representative FRET traces (transitions shown in red) and corresponding histograms of the docking equilibrium in the absence of CYT-19 (top) and with 1 µM CYT-19 and 2 mM ATP-Mg2+ (bottom) for a P1 helix formed with the oligonucleotide −1 m,rSA3C2 (see Table S3). (B) Lifetime plots for docked and undocked P1 in the absence of CYT-19 (black) and with 1 µM CYT-19 and 2 mM ATP-Mg2+ (blue). See also Data S1. Values of the docking rate and equilibrium constants are shown in Table S1 for this helix and a second helix that docks weakly (formed with −3 m,rSA3C2; see Table S3). (TIF) [file pbio.1001981.s005.tif]

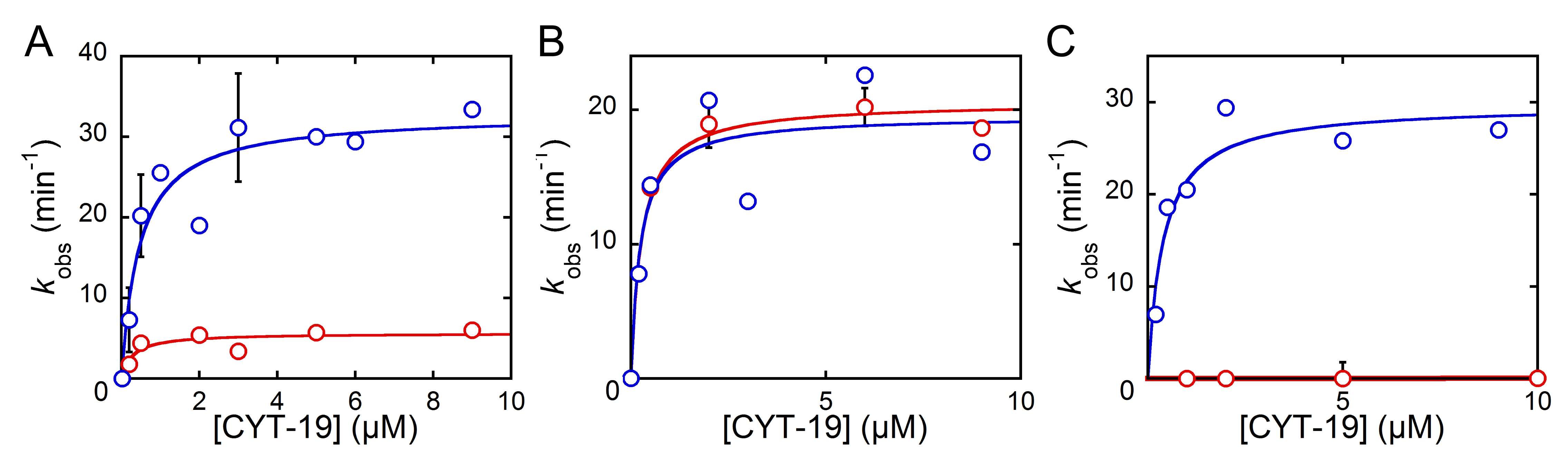

Supplement: Figure S6 — CYT-19–mediated unwinding of the 6-bp P1 helix is rate limited by spontaneous undocking of P1. To verify that the observed correlation between the maximum P1 unwinding rate and the undocking rate is due to P1 docking stability, ensemble experiments were performed with the native Tetrahymena ribozyme and its long-lived misfolded conformer, which does not stably dock the P1 helix [29]. See Table S3 for sequences and properties of substrate oligonucleotides. (A) The CYT-19 concentration dependence for unwinding the 6-bp P1 helix formed with substrate −1 d,rSA5 by the native ribozyme shows a maximum unwinding rate constant (k max) of 6 min−1 (red), which is comparable to the intrinsic undocking rate constant measured in single molecule experiments (Figure 2C, top and Table S1). When docking is inhibited by misfolding the ribozyme (blue), k max is increased to ∼30 min−1. (B) With a substrate for which P1 docking is inhibited by replacement of a 2′-hydroxyl group with a 2′-O-methyl group (−3 m,rSA5), the undocked state predominates and CYT-19–mediated unwinding is accelerated, with no difference between the native ribozyme (red) and the misfolded ribozyme (blue). We infer that the lower value for the k max of this substrate compared to the standard substrate (−1 d,rSA5, Figure S6A) reflects an effect of the methoxy substitution on CYT-19–mediated unwinding. (C) CYT-19–mediated unwinding of the P1 duplex containing the 6-nt product (rP), which docks much more strongly than the helix formed with the standard substrate. As above, results from the native and misfolded ribozyme species are shown in red and blue, respectively. Error bars represent the standard deviation of at least two independent measurements. (TIF) [file pbio.1001981.s006.tif]

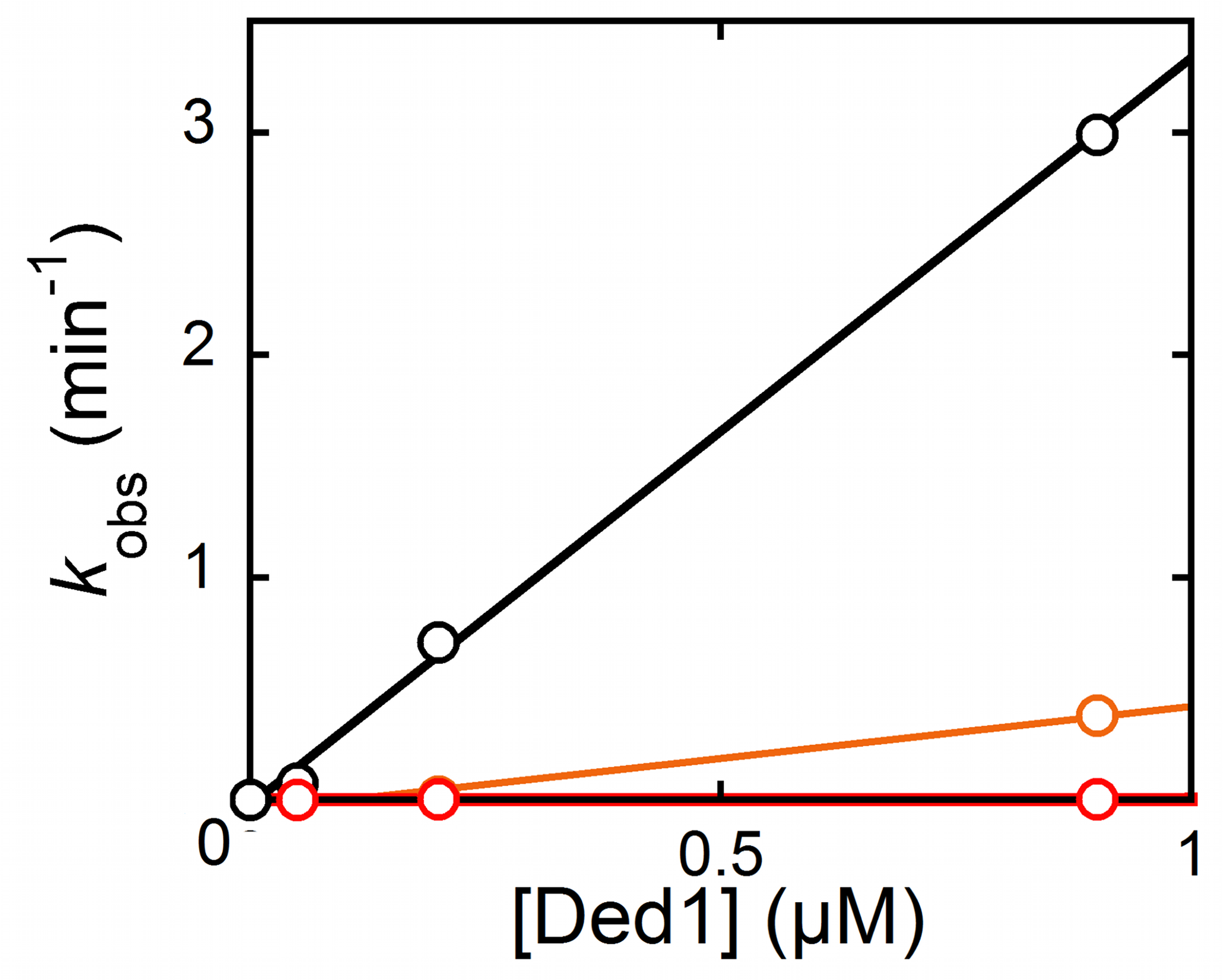

Supplement: Figure S7 — Unwinding of the standard 11-bp P1 helix by Ded1. Ded1 unwinds P1 in the presence of 2 mM ATP with a second order rate constant of 3.4×10^6^ M−1·min−1 (black). Secondary structure disruption by Ded1 is reduced in the presence of 2 mM AMP–PNP (orange, 4.6×10^5^ M−1·min−1), and without nucleotide (red, 2.8×10^3^ M−1·min−1). (TIF) [file pbio.1001981.s007.tif]
