# Supplementary material for: DEAD-Box Helicase Proteins Disrupt RNA Tertiary Structure Through Helix Capture
Source: PLoS Biol. 2014 Oct 28;12(10):e1001981. doi: 10.1371/journal.pbio.1001981 (PMC4211656; doi:10.1371/journal.pbio.1001981)
Supplement: Table S1 — P1 docking kinetics and equilibria for the 11-bp P1 helix. Values were determined in single molecule fluorescence experiments except where indicated. The slow phase for P1 docking in the absence of CYT-19 is attributed to heterogeneous P1 docking behavior (Figure S2). The docking rate constant in the presence of CYT-19 (k dock) was calculated as described in Text S1 (“Determination of P1 Docking and Undocking Kinetics”). Except where indicated, the observed rate constant for unwinding (k obs,unwind) was determined by single molecule fluorescence by monitoring the disappearance of substrate from the ribozyme over time, using multiple fields of view. Thus, k obs,unwind reflects the overall rate constant for the two-step process of undocking and helix unwinding. See Table S3 for sequences and effects of each substrate. See also Data S1. aRelative amplitudes for each phase of the docking kinetics were determined from the fit of the undocked lifetimes normalized by total number of transition events and are listed in parentheses. bRate constants for P1 unwinding in the absence of CYT-19 were measured in ensemble experiments. (DOCX) [file pbio.1001981.s008.docx]

Table S1.

| **substrate** | **[CYT-19] (μM)** | **nucleotide** | **# molecules** | ***K*_dock_** | ***k*_undock_ (min^-1^) (amp)^a^** | **τ_undocked state_ (s) (amp)^a^** | ***k*_dock_ (min^-1^)** | ***k*_obs,unwind_ (min^-1^)** |
| --- | --- | --- | --- | --- | --- | --- | --- | --- |
| -1d,rSA_3_C_2_ | 0 |  | 2439 | 8.4 | 20 | 0.52 (0.92)  4.3 (0.08) | 120 | 0.0004^b^ |
|  | 0.5 | ATP | 952 | 4.7 | 22 | 0.55 (0.62)  3.0 (0.38) | 7.7 | 0.16±0.02 |
|  | 0.5 | AMP-PNP | 546 | 4.5 | 17 | 0.56 (0.86)  2.9 (0.14) | 8.6 | 0.007 |
|  | 1 | ATP | 209 | 2.4 | 17 | 0.53 (0.58)  4.0 (0.42) | 4.0 | 0.50±0.13 |
|  | 1 | AMP-PNP | 877 | 2.8 | 17 | 0.50 (0.37)  4.2 (0.63) | 3.6 | 0.07±0.03 |
|  | 1 | ADP | 244 | 2.3 | 25 | 0.50 (0.86)  2.9 (0.14) | 15 | 0.005 |
|  | 1 | no nt | 189 | 1.7 | 22 | 0.53 (0.82)  3.2 (0.18) | 10 | 0.014 |
|  | 2 | ATP | 217 | 0.99 | 18 | 0.52 (0.54)  3.2 (0.46) | 4.0 | 0.78±0.09 |
|  | 2 | ADP | 206 | 4.1 | 16 | 0.43 (0.47)  2.7 (0.53) | 9.3 | 0.03 |
|  | 2 | no nt | 307 | 2.1 | 16 | 0.47 (0.63)  3.3 (0.37) | 9.9 | 0.06 |
| -1m,rSA_3_C_2_ | 0 | ATP | 293 | 0.54 | 150 | 0.62 | 97 | 0.0013^b^ |
|  | 1 | ATP | 120 | 0.14 | 180 | 0.73 (0.58)  2.0 (0.42) | 19 | 0.44 |
| -3m,rSA_3_C_2_ | 0 | ATP | 111 | 0.22 | 190 | 0.79 | 76 |  |
|  | 2 | ATP | 170 | 0.03 | 204 (0.98)  4.8 (0.02) | 0.80 (0.90)  3.8 (0.10) | 6.5 | 0.63 |
