# Supplementary material for: DEAD-Box Helicase Proteins Disrupt RNA Tertiary Structure Through Helix Capture
Source: PLoS Biol. 2014 Oct 28;12(10):e1001981. doi: 10.1371/journal.pbio.1001981 (PMC4211656; doi:10.1371/journal.pbio.1001981)
Supplement: Table S2 — Rate constants of the various “fates” of undocked P1 helix. In the presence of CYT-19, the undocked P1 helix may redock or unwind. Additionally, the fluorescence signal may be artificially truncated by the shuttering of the excitation laser. For each CYT-19 concentration, the fractions of undocking events that ended with redocking, unwinding, or were truncated by the shutter were determined and the corresponding rate constants (k dock, k unwind, and k truncation, respectively) were calculated by multiplying the observed rate constant (k obs) by the probabilities of each outcome (see Text S1, “Determination of P1 Docking and Undocking Kinetics” for details). To determine the unwinding rate constant (k unwind), the calculated rate constant reflecting disappearance of Cy3 was further corrected by subtracting the rate constant for Cy3 photobleaching, as measured independently (k photobleach = 0.55 min−1; Figure S1). Values reported in the text as the fraction of events that ended in unwinding or redocking express these outcomes relative to each other—that is, normalized to 100%. (DOCX) [file pbio.1001981.s009.docx]

Table S2.

|  |  | Redocking | Unwinding | Truncation by shutter | Total |
| --- | --- | --- | --- | --- | --- |
| **[CYT-19] (µM)** | **# molecules** | ***k*_dock_ (min^-1^) (fraction)** | ***k*_unwind_ (min^-1^) (fraction)** | ***k*_truncation_ (min^-1^) (fraction)** | ***k*_obs_ (min^-1^) (fraction)** |
| 0.5 | 945 | 7.7 (0.38) | 6.8 (0.37) | 4.9 (0.25) | 20 (1) |
| 1 | 206 | 4.0 (0.27) | 3.3 (0.25) | 7.2 (0.48) | 15 (1) |
| 2 | 217 | 4.0 (0.21) | 9.7 (0.55) | 4.5 (0.24) | 19 (1) |
