# Supplementary material for: DEAD-Box Helicase Proteins Disrupt RNA Tertiary Structure Through Helix Capture
Source: PLoS Biol. 2014 Oct 28;12(10):e1001981. doi: 10.1371/journal.pbio.1001981 (PMC4211656; doi:10.1371/journal.pbio.1001981)
Supplement: Table S3 — Sequences and properties of oligonucleotides used in ensemble and single molecule experiments. In order for the P1 helix to be visualized with smFRET, the indicated oligonucleotides were labeled on their 3′-end with Cy3 dye and the DNA tether was labeled with its FRET pair, Cy5. For the first two oligonucleotides, Cy3 replaces the 3′ nucleotide (i.e., resulting in −1 d, rSA4-Cy3 and −3 m,rSA4-Cy3). (DOCX) [file pbio.1001981.s010.docx]

Table S3.

| **oligonucleotide** | **properties** | **sequence** |
| --- | --- | --- |
| -1d,rSA_5_ | minimizes 5’ cleavage | CCCUCdUAAAAA (with and without 3’ Cy3) |
| -3m,rSA_5_ | disrupts P1 docking | CCCmUCUAAAAA (with and without 3’ Cy3) |
| rP | strengthens P1 docking | CCCUCU |
| -1d,rSA_3_C_2_ | minimizes 5’ cleavage | CCCUCdUAAACC (with and without 3’ Cy3) |
| -1m,rSA_3_C_2_ | destabilizes P1 docking | CCCUCmUAAACC (with and without 3’ Cy3) |
| -3m,rSA_3_C_2_ | destabilizes P1 docking | CCCmUCUAAACC (with and without 3’ Cy3) |
| tether-Cy5 | immobilizes ribozyme to slide | biotin-d(TGTGTAAGTTTTAGGTTGATTTTG)-Cy5 |
