# Supplementary material for: DEAD-Box Helicase Proteins Disrupt RNA Tertiary Structure Through Helix Capture
Source: PLoS Biol. 2014 Oct 28;12(10):e1001981. doi: 10.1371/journal.pbio.1001981 (PMC4211656; doi:10.1371/journal.pbio.1001981)
Supplement: Table S4 — Single molecule observation of Tetrahymena ribozyme with the 6-bp P1 before and after addition of 10 nM CYT-19. Prior to CYT-19 addition, each field of view on the slide showed an average of 17 molecules (from three FOVs), as indicated. Upon CYT-19 addition, the number of visible molecules decreased and remained constant, as expected based on the P1 unwinding rate constant measured under the same conditions in ensemble experiments [17]. The number of molecules for each time point shown was determined for different FOVs on the slide to minimize the contribution of dye photobleaching. The low number of ribozyme molecules observed per FOV after the addition of CYT-19 and the lack of a detectable time dependence prevented a robust analysis of the time dependence or docking dynamics for this shorter P1 helix. (DOCX) [file pbio.1001981.s011.docx]

| **Time after addition of 10 nM CYT-19 (min)** | **# molecules per FOV** |
| --- | --- |
| 0 (buffer) | avg ~ 17±4.7 |
| 1.6 | 10 |
| 3 | 4 |
| 4 | 17 |
| 5.3 | 6 |
| 7.3 | 8 |
| 8.8 | 8 |
| 24.8 | 3 |
| 26.3 | 1 |
| 27.3 | 7 |
| 29.5 | 5 |
