# Supplementary material for: DEAD-Box Helicase Proteins Disrupt RNA Tertiary Structure Through Helix Capture
Source: PLoS Biol. 2014 Oct 28;12(10):e1001981. doi: 10.1371/journal.pbio.1001981 (PMC4211656; doi:10.1371/journal.pbio.1001981)
Supplement: Table S5 — Docking kinetics for the 11-bp P1 helix as measured by single molecule fluorescence in the presence of Ded1 and the indicated nucleotides. The docking rate constant in the presence of Ded1 (k dock) was calculated as for CYT-19 (see Text S1, “Determination of P1 Docking and Undocking Kinetics”). The rate constant for P1 unwinding by Ded1 (k uw) was also calculated as described for CYT-19 and determined to be 5.5±2.1 min−1. See also Data 1. aAmplitudes for each phase of the docking kinetics, listed in parentheses, were determined from the fit of the undocked lifetimes and normalized by the total number of transition events. (DOCX) [file pbio.1001981.s012.docx]

Table S4.

| **[Ded1] (µM)** | **nucleotide** | **# molecules** | ***k*_dock_ (min^-1^) (amp)^a^** | ***k*_undock_ (min^-1^)** |
| --- | --- | --- | --- | --- |
| 0.05 | ATP | 292 | 11 (0.27)  160 (0.73) | 16 |
| 0.1 | AMP-PNP | 143 | 20 (0.14)  109 (0.86) | 18 |
| 0.1 | no nt | 137 | 105 | 17 |
| 0.2 | ATP | 398 | 20 (0.20)  120 (0.80) | 19 |
| 0.9 | AMP-PNP | 323 | 20 (0.23)  130 (0.77) | 24 |
| 0.9 | no nt | 125 | 116 | 16 |
