# Supplementary material for: DEAD-Box Helicase Proteins Disrupt RNA Tertiary Structure Through Helix Capture
Source: PLoS Biol. 2014 Oct 28;12(10):e1001981. doi: 10.1371/journal.pbio.1001981 (PMC4211656; doi:10.1371/journal.pbio.1001981)
Supplement: Text S1 — Single molecule data analysis, including descriptions of molecule selection, determination of P1 docking and undocking kinetics, and P1 unwinding as monitored by single molecule fluorescence. (DOC) [file pbio.1001981.s015.doc]

**SUPPORTING TEXT S1: Single molecule data analysis**

**Selection of molecules**

For each data acquisition, fluorescent ‘spots’ of interest within each field of view were identified by mapping pixels from the “red” side to the “green” side based on an affine transformation determined from images using fluorescent beads as fiducial markers. Local background was subtracted from each selected spot for each frame.

Fluorescence intensity traces were first automatically chosen based on intensity thresholds and then inspected visually. Single RNA molecules were selected based on fulfillment of the following criteria: (1) the presence of active Cy5, based on average fluorescence intensity under the red laser or the presence of a high FRET state under green laser, (2) the presence of active Cy3, based on average fluorescence intensity under green laser, (3) stable signal strength, indicating a single molecule. Approximately 65% of automatically selected traces in each field of view were ultimately selected for further analysis. FRET traces for these molecules were calculated from the recorded fluorescence intensities of the Cy3 and Cy5 dyes with 9-pt smoothing applied.

$\text{FRET}=\frac{(\text{I}_{\text{Cy5}}-\beta\times\text{I}_{\text{Cy3}})}{(\text{I}_{\text{Cy3}}+\text{I}_{\text{Cy5}})}$ ,

where *β* represents the degree of Cy3 emission that crosses over to the red channel.

**Determination of P1 docking and undocking kinetics**

Transitions between two FRET states (high FRET ~0.9 and low FRET ~0.2), representing docked and undocked states, were observed for these ribozyme molecules as previously described [1,2]. Thresholds were set from 0 to 0.5 for detection of the low FRET state and from 0.7 to 1 for the high FRET state. The docking equilibrium constant (*K*_dock_) was determined from the ratio of total time spent in the docked and undocked states as calculated from the Gaussian fit of each peak in the FRET histogram. Time constants for undocked and docked P1 (τ_undocked_ and τ_docked_, respectively) were calculated using the dwell times in the low and high FRET states, respectively [1]. The corresponding rate constants for most molecules under CYT-19 buffer conditions (see above), were well-described by a single-exponential fit. However, taken as an ensemble, heterogeneity in intrinsic docking behavior contributed to a small second phase (Figure S2 and Table S1).

The lifetime of the docked state, τ_docked_, was not changed significantly by CYT-19 or Ded1. However, the lifetime in the undocked state became biphasic, with a fraction of events having a larger τ_undocked_, i.e. a slower than intrinsic observed docking rate constant (*k*_obs_). Several parallel processes contribute to *k*_obs_: (1) redocking, (2) unwinding of P1, (3) photobleaching of Cy3, and (4) truncation of the time trace. That is,

$k_{\text{obs}}=k_{\text{dock}}+k_{\text{unwind}}+k_{\text{Cy3} \text{photobleach}}+k_{\text{truncation}}$ .

The rate constants for each of these processes in the presence of CYT-19 or Ded1 were determined by multiplying *k*_obs_ by the relative probability (F) of each “fate” for those events that contribute to the slow phase. These long undocked events were defined as having a minimum lifetime of ~2 s for CYT-19 and ~1 s for Ded1 (these lifetimes are based on the double exponential fit of the cumulative number of undocked events). For example, the docking rate constant for P1 in the presence of either CYT-19 or Ded1 (*k*_dock_) was calculated by determining the fraction of these long-lived undocked events that resulted in redocking of P1 (F_docked_, see below). For our RNA construct, the signals for P1 unwinding and Cy3 photobleaching are the same. Therefore, both processes are included in the calculation of F_unwound/PB_ (see below).

$$\begin{matrix} \text{F}_{\text{docked}}=\frac{\text{N}_{\text{docked}}}{\text{N}_{\text{undocked}}} , \\ \text{F}_{\text{unwound/PB}}=\frac{\text{N}_{\text{unwound/PB}}}{\text{N}_{\text{undocked}}} , \\ \text{F}_{\text{truncated}}=\frac{\text{N}_{\text{truncated}}}{\text{N}_{\text{undocked}}} , \end{matrix}$$

where N_undocked_ is the total number of long undocked events and N_docked_ is the number of these transition events that dock after a long dwell time (see above) in the undocked state. Likewise, N_unwound/PB_ is the number of P1 unwinding and Cy3 photobleaching events, and N_truncated_ is the number of long undocked states that are limited by the observation time. The rate constant for Cy3 photobleaching (*k*_photobleach_) was measured independently (0.55 min^–1^, Figure S1) and subtracted from *k*_unwind_.

$$\begin{matrix} k_{\text{dock}}=\text{F}_{\text{docked}}\times k_{\text{obs}} , \\ k_{\text{unwind}}=(\text{F}_{\text{unwound/PB}}\times k_{\text{obs}})-k_{\text{photobleach}} , \\ k_{\text{truncation}}=\text{F}_{\text{truncated}}\times k_{\text{obs}} . \end{matrix}$$

The results for CYT-19-dependent *k*_dock_ are shown in Tables S1 and S2. The average *k*_dock_ in the presence of CYT-19 and ATP is 5.2±2.1 min^–1^ (Table S2). Calculations were performed with an observation time of 10 s. Data for longer (~15-30 s) observation times yielded similar results (within 2-3-fold, not shown). Results for *k*_unwind_ are shown in Table S2 and reflect unwinding of P1 by CYT-19 from the undocked conformation. The results for Ded1 are shown in Table S5.

**P1 unwinding monitored using single molecule fluorescence**

P1 unwinding by CYT-19 was measured by single molecule fluorescence by monitoring the intact P1 helices over time. Data were taken over multiple fields of view and the number of Cy3-labeled P1 helices was normalized by the amount of Cy5-tether within the same field of view. Rate constants for unwinding by CYT-19 determined this way are shown in Table S1 and are within 3-5-fold of benchtop results (Figure S3).

While CYT-19 increased P1 unwinding from the low FRET state, a fraction of unwinding events appeared to occur from the high FRET state (~20% of all molecules). The frequency of these events, however, was not affected significantly by the presence of CYT-19 or Ded1, indicating that this behavior was not a protein-dependent effect and most likely reflected photophysical processes [3–5].

**SUPPLEMENTARY REFERENCES**

1. Bartley LE, Zhuang X, Das R, Chu S, Herschlag D (2003) Exploration of the transition state for tertiary structure formation between an RNA helix and a large structured RNA. J Mol Biol 328: 1011–1026.

2. Solomatin SV, Greenfeld M, Chu S, Herschlag D (2010) Multiple native states reveal persistent ruggedness of an RNA folding landscape. Nature 463: 681–684. doi:10.1038/nature08717.

3. Levitus M, Ranjit S (2011) Cyanine dyes in biophysical research: the photophysics of polymethine fluorescent dyes in biomolecular environments. Q Rev Biophys 44: 123–151. doi:10.1017/S0033583510000247.

4. Iqbal A, Arslan S, Okumus B, Wilson TJ, Giraud G, et al. (2008) Orientation dependence in fluorescent energy transfer between Cy3 and Cy5 terminally attached to double-stranded nucleic acids. Proc Natl Acad Sci U S A 105: 11176–11181. doi:10.1073/pnas.0801707105.

5. Iqbal A, Wang L, Thompson KC, Lilley DM, Norman DG (2008) The structure of cyanine 5 terminally attached to double-stranded DNA: implications for FRET studies. Biochemistry 47: 7857–7862. doi:10.1021/bi800773f.
