# Supplementary material for: DEAD-Box Helicase Proteins Disrupt RNA Tertiary Structure Through Helix Capture
Source: PLoS Biol. 2014 Oct 28;12(10):e1001981. doi: 10.1371/journal.pbio.1001981 (PMC4211656; doi:10.1371/journal.pbio.1001981)
Supplement: Text S2 — Supplementary methods, including purification of CYT-19 and slide preparation for single molecule experiments. (DOC) [file pbio.1001981.s016.doc]

**SUPPORTING TEXT S2: Supplementary Methods**

**CYT-19 purification**

CYT-19 was expressed from *Escherichia coli* strain BL-21 as a fusion with maltose-binding protein (MBP). Induction was performed at 20 °C for 16 h in the presence of 1 mg/ml isopropyl-b-D-thiogalactopyranoside (IPTG). Cells were harvested by centrifugation, resuspended in amylose buffer (20 mM Tris-HCl, pH 7.5, 1 mM EDTA, 2 mM dithiothreitol (DTT), 10% glycerol) containing 0.5 M KCl, and lysed by incubating with 1 mg/ml lysozyme on ice for 20 min, followed by three 15 s sonication bursts (power setting 6 with a double-stepped microtip; Branson Sonifier S-450-A; VWR Scientific). The lysate was cleared by centrifugation (18,500 × g for 30 min at 4 °C). Polyethyleneimine (PEI) was added slowly to 0.4% and particulates were filtered out (0.2 µm cellulose acetate membrane). The solution was loaded onto a 5-ml amylose column (high-flow resin; New England Biolabs) and washed with 5 column volumes (CV) of amylose buffer containing 0.5 M KCl. The column was then washed with 10 CV of amylose buffer containing 1.5 M KCl and washed again with 10 CV of amylose buffer containing 0.5 M KCl. The fusion protein was eluted with amylose buffer containing 10 mM maltose. Peak fractions were pooled and incubated overnight at 4 °C in the presence of 40 mg/ml tobacco etch virus (TEV) protease to cleave the MBP tag. To remove the cleaved tag, the preparation was diluted to 0.3 M KCl and loaded on a 2-ml heparin column (GE). The heparin column was washed with 10 CV of 20 mM Tris (pH 7.5), 0.3 M KCl, 1 mM EDTA and 2 mM DTT. CYT-19 was eluted with a salt gradient (0.3 M KCl to 1 M KCl). Peak fractions were dialyzed (separately) overnight at 4 °C against 50 volumes of storage buffer (20 mM Tris-HCl (pH 8.5), 0.5 M KCl, 1 mM EDTA, 0.2 mM DTT, 50% (v/v) glycerol) and stored at -80 °C.

**Slide preparation for single molecule experiments**

Quartz slides were incubated in 10% Alconox solution overnight. After a brief sonication (1 min) and thorough rinsing with ePure water, slides were incubated in a solution of NoChromix (Godax Laboratories) and sulfuric acid for at least 24 hours. They were then cleaned by successive 20-min sonications in acetone, ethanol and 1 M KOH with thorough rinsing with ePure water between each step. Slides were then pyrolyzed before a 10-min sonication in methanol in preparation for aminosilanization. Glass coverslips were sonicated in 1 M KOH (20 min) and then in methanol (10 min) before aminosilanization.

Cleaned slides and coverslips were incubated for 10 min in a methanol solution of 1% aminosilane (United Chemical Technologies) and 5% acetic acid (Fisher Scientific), sonicated for one min and then incubated for another 10 min in the same solution before being rinsed with methanol and ePure water, and dried with nitrogen. Coverslips and slides were then incubated for at least three hours in a 40:1 solution of methoxy-poly(ethylene glycol)-succinimidyl valerate (5000 kD mPEG-SVA, Laysan Bio) and biotin-poly(ethylene glycol)-succinimidyl valerate (5000 kD biotin-PEG-SVA, Laysan Bio) made in 100 mM sodium bicarbonate buffer (pH 8.5). Coverslips and slides were then rinsed thoroughly with ePure water, dried with nitrogen and stored in the dark at -20 °C.
